# Chiral domain dynamics and transient interferences of mirrored superlattices in nonequilibrium electronic crystals

**DOI:** 10.1038/s41598-023-46659-y

**Published:** 2023-11-10

**Authors:** J. Ravnik, Ye. Vaskivskyi, J. Vodeb, M. Diego, R. Venturini, Ya. Gerasimenko, V. Kabanov, A. Kranjec, D. Mihailovic

**Affiliations:** 1 Complex Matter Department, Jozef Stefan Institute, Jamova 39, 1000 Ljubljana, Slovenia; 2 Laboratory for Micro and Nanotechnology, Paul Scherrer Institut (PSI), Forschungsstrasse 111, 5232 Villigen, Switzerland; 3 Center of Excellence on Nanoscience and Nanotechnology-Nanocenter (CENN Nanocenter), Jamova 39, 1000 Ljubljana, Slovenia; 4 Faculty for Mathematics and Physics, University of Ljubljana, Jadranska 19, 1000 Ljubljana, Slovenia

**Keywords:** Electronic properties and materials, Physics, Quantum fluids and solids

## Abstract

Mirror symmetry plays a major role in determining the properties of matter and is of particular interest in condensed many-body systems undergoing symmetry breaking transitions under non-equilibrium conditions. Typically, in the aftermath of such transitions, one of the two possible broken symmetry states is emergent. However, synthetic systems and those formed under non-equilibrium conditions may exhibit metastable states comprising of both left (L) and right (R) handed symmetry. Here we explore the formation of chiral charge-density wave (CDW) domains after a laser quench in 1T-TaS_2_ with scanning tunneling microscopy. Typically, we observed transient domains of both chiralities, separated spatially from each other by domain walls with different structure. In addition, we observe transient density of states modulations consistent with interference of L and R-handed charge density waves within the surface monolayer. Theoretical modeling of the intertwined domain structures using a classical charged lattice gas model reproduces the experimental domain wall structures. The superposition (S) state cannot be understood classically within the correlated electron model but is found to be consistent with interferences of L and R-handed charge-density waves within domains, confined by surrounding domain walls, vividly revealing an interference of Fermi electrons with opposite chirality, which is not a result of inter-layer interference, but due to the interaction between electrons within a single layer, confined by domain wall boundaries.

## Introduction

The hexagonal atomic lattice, which is common in layered 2D materials, supports commensurate (C) electronic superlattice ordering with various sizes of unit cell. Experimentally, the size of the superlattice unit cell has been observed in the range between 3 to 16 lattice constants, and values in between, which are allowed by the geometrical cosine rule^1^. Some of these superlattices break crystal mirror symmetry, which occurs when the superlattice ordering vector is not aligned with a symmetry axis of the atomic lattice. Such superlattices can exist in two possible chiralities, and we can transform one into the other by a mirroring operation over a high symmetry axis of the atomic lattice. If we write the superlattice unit vector as a combination of the in-plane atomic unit cell vectors ${\varvec{a}}$ and ${\varvec{b}}$ as $n{\varvec{a}}+m{\varvec{b}}$, then this condition is satisfied when $n\ne m$ and $n,m>0$. Examples of such superlattices are described by unit cells such as $\sqrt{7}\times \sqrt{7}$, $\sqrt{13}\times \sqrt{13}$ and $\sqrt{21}\times \sqrt{21}$. The $\sqrt{13}\times \sqrt{13}$ superlattice is commonly found in nature^1^, and appears in various materials, including 1T-TaS_2_, 2Hb-TaS_2_, 1T-TaSe_2_ and 2Hb-TaSe_2_^2^. As a special case, also the $\sqrt{7}\times \sqrt{7}$ superstructure has been observed in doped surface layers of TaS_2_^3^. Energetically, the L and R-handed superlattices are equivalent^4^. A mixture of the two would result in domain walls between them, which is energetically costly^5,6^ and thus coexisting mirrored domains are experimentally rarely found under equilibrium conditions. The presence of mirrored domains was nevertheless observed in the scattering experiments^7^, but it was not investigated in detail until recently^8–10^. The scattering experiments are in this case limiting, as they simultaneously probe many layers as well as a relatively large area within each layer, and thus give little or no information on the microscopic ordering, making it unclear whether the L and R domains exist within a single layer or across many layers. Recently Ohta et al. investigated stacking of L and R domains in Nb substituted 1T-TaSe_2_, reporting different electronic states due to the different stackings^11^.

In non-equilibrium systems, however, various metastable states may be observable. It was recently shown by Zong et al. that applying an ultrafast laser pulse to a thin flake of 1T-TaS_2_ leads to the appearance of the symmetrically equivalent CDW diffraction peaks which were attributed to the mirrored superlattice orientations^12^, whereby the probability of switching increases with increasing the laser fluence. After the excitation, both diffracted peaks were present in regions of the size of 1.1 μm. Due to the experimental limitations, it was not clear whether this is an interlayer or an intralayer effect, and how the domains order and interact. In addition to mixed L and R superlattice states, an intriguing possibility exists in quantum systems. Considering that L and R configurational states are energetically degenerate, a superposition (S) of the L and R state is also possible. While CDWs are commonly described as classical objects, in which superpositions can occur, properties such as CDW tunneling reveal their quantum nature^13–15^. In the quantum description, the CDW can be thought of in terms of a standing wave, arising from an interference of counter-propagating electrons with wavevector ${k}_{CDW}$. However, interference of CDWs with different in-plane chiralities within a single layer without proximity of adjacent layers has to our knowledge not been observed until now.

Using scanning tunneling microscopy, we investigate the microscopic structure of the mirror domains in the surface layer of 1T-TaS_2_ created by laser excitation in the low-temperature C state. The STM images show the local density of states (LDOS), which in the quasiparticle approximation is given by: ${\rho }_{QP}\left(E,\mathbf{r}\right)\propto {\Sigma }_{k}{\left|{\uppsi }_{k}\left(\mathbf{r}\right)\right|}^{2}\delta (E-\varepsilon (\mathbf{k})$), where $\varepsilon \left(\mathbf{k}\right)$ is the energy of the electrons with different wavevector $\mathbf{k}$ that interfere locally at position $\mathbf{r}$, allowing us to measure interferences of CDWs. Such quasiparticle interferences surrounding an impurity were previously observed in cuprate superconductors^11^, and for confined electron trajectories in transition metal dichalcogenide heterostructures^12^. The observations by STM are classical, but the observed quasiparticle interference phenomenon is treated by solving the Schrödinger equation for counterpropagating quasiparticles. Since the observed order parameter does not have any out-of-plane component (STM scans are 2D and surface sensitive), we primarily consider the system as two-dimensional, where CDWs tilted L and R with respect to an in-plane crystal axis are considered two different chiralities.

**Figure 1:**
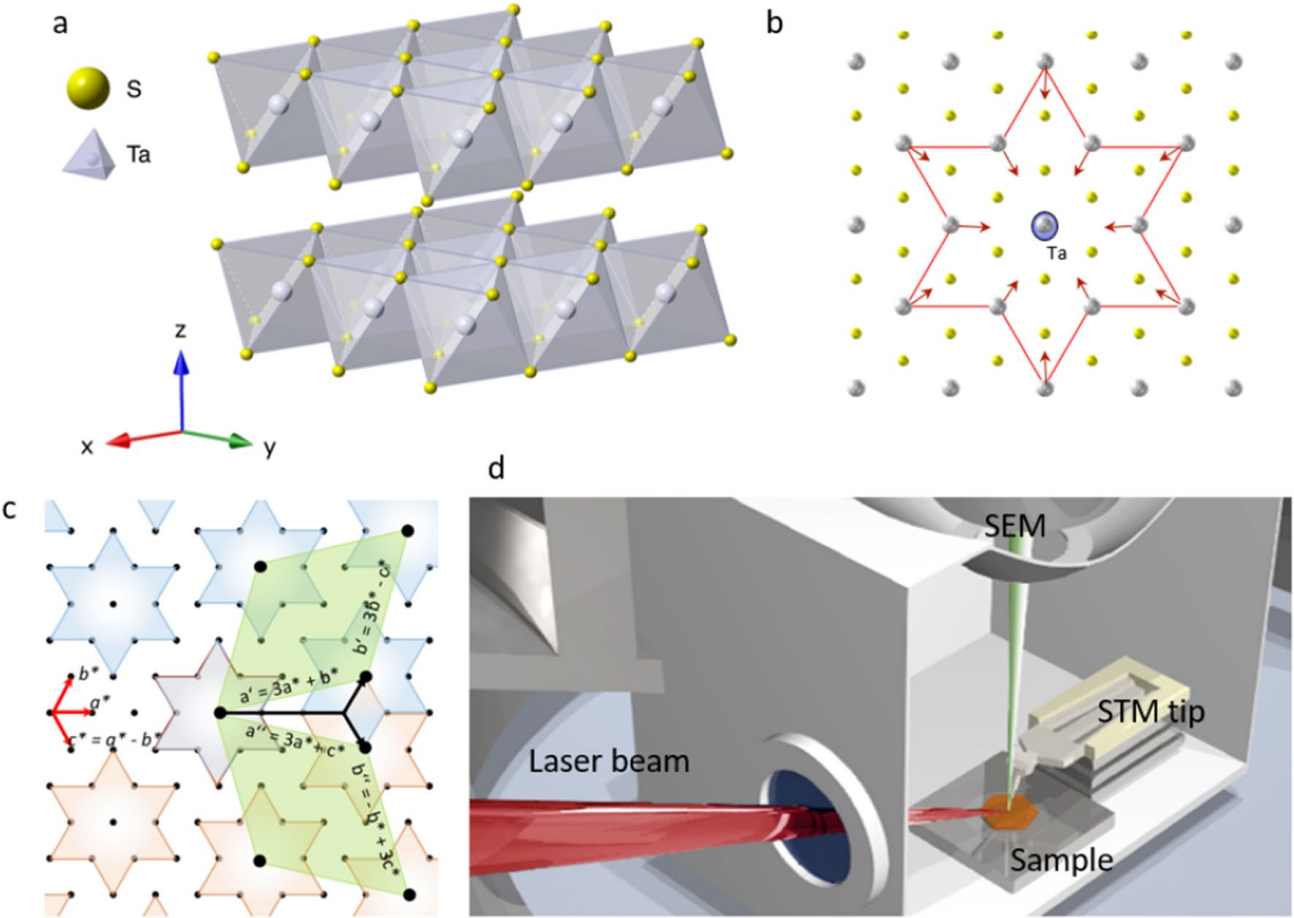
(**a**) Crystal structure of 1T-TaS_2_. (**b**) A schematic representation of the polaron structure formed within the $\sqrt{13}\times \sqrt{13}$ superlattice. (**c**) The commensurate $\sqrt{13}\times \sqrt{13}$ superlattice can take two different orientations, rotated by $\pm$ 13.9° with respect to the atomic lattice. (**d**) A schematic diagram of the experimental setup.

1T-TaS_2_ is a quasi-two-dimensional transition metal dichalcogenide, where tantalum atoms are sandwiched between two layers of Sulphur atoms (Fig. 1a). The layers are stacked together in the z-direction to form a highly anisotropic quasi-two-dimensional crystal. At temperatures above 550 K, the material is in a metallic state. Below 550 K an incommensurate (IC) charge density wave appears, whose modulation vectors are aligned with the lattice direction, thus keeping the in-plane mirror symmetry intact, and breaking only translational lattice symmetry^16^. A mirror symmetry breaking transition to a nearly commensurate (NC) CDW state occurs at 350 K, where the direction of the charge modulation is misaligned with the lattice by +$11^\circ$ or −$11^\circ$, as observed in diffraction or STM experiments^2,17^. Below 180 K, the CDW becomes commensurate (C) and insulating^18^. Here, the angle between the $\sqrt{13}\times \sqrt{13}$ superlattice direction and the crystal lattice direction is about ${\theta }_{S}=13.9$ degrees as defined by the geometry of electron ordering on a triangular lattice^4^. The superlattice unit cell is formed with 13 atoms, where twelve of the atoms move towards the thirteenth central atom to form a David-star-like cluster, as shown in Fig. 1b. In order for these clusters to cover the whole plane, two different tiling configurations are possible with angles at ± 13.9° with respect to the atomic lattice (Fig. 1c). It was recently shown that we can use optical or electrical pulses to induce a metallic hidden (H) state^19–22^. STM studies of the surface show that the uniform CDW pattern is broken into smaller domains. The domains and domain walls are metastable at low temperatures, which allows us to investigate their non-periodic microscopic order by STM^21,22^. The H state formation is macroscopically a very reproducible phenomenon, as shown by electrical and optical experiments^19,23,24^, but the microscopic domain distribution appears to be different after each switching attempt^24^, which is in line with the probabilistic nature of the appearance of the mirror domains at higher temperatures^12^. X-ray and electron diffraction experiments^25,26^ show that interlayer stacking plays a role in ordering within the H state, but do not reveal any domain structure. STM observations on the other hand reveal that long-range order of the surface layers is broken in the H state. With STM we only probe the top layer and thus observe only the ordering along the h and k axis (parallel to the layers), while it is not possible to probe the out-of-plane dimension and thus hard to reach conclusions about the bulk. An important thing to note is that in three dimensions, the CDW state can have 4 possible orientations, as in addition to the symmetry within the *h*–*k* plane, we must also consider the mirroring over the *h*–*k* plane, thus negating the *l* component of the CDW. Since the observed order parameter does not have any out-of-plane component, we can distinguish only the difference within the *h*–*k* plane, while the symmetric orderings with respect to the *l* direction appear identical. In this sense we consider the system as two-dimensional, justifying the use of the term chirality to describe L and R domains.

Apart from finding a rich variety of domain walls between L and R domains, that are not observed in equilibrium crystals, we also observe metastable domains where the local density of states shows complex flower-shaped spatial patterns, in which twofold symmetry axis is restored. We show that the symmetry and observed patterns can be understood in terms of superpositions of L and R states. We successfully model the domain walls using a charged lattice gas (CLG) model, while the complex interference patterns are modelled in terms of superpositions of L and R CDWs.

## Experimental results

**Figure 2:**
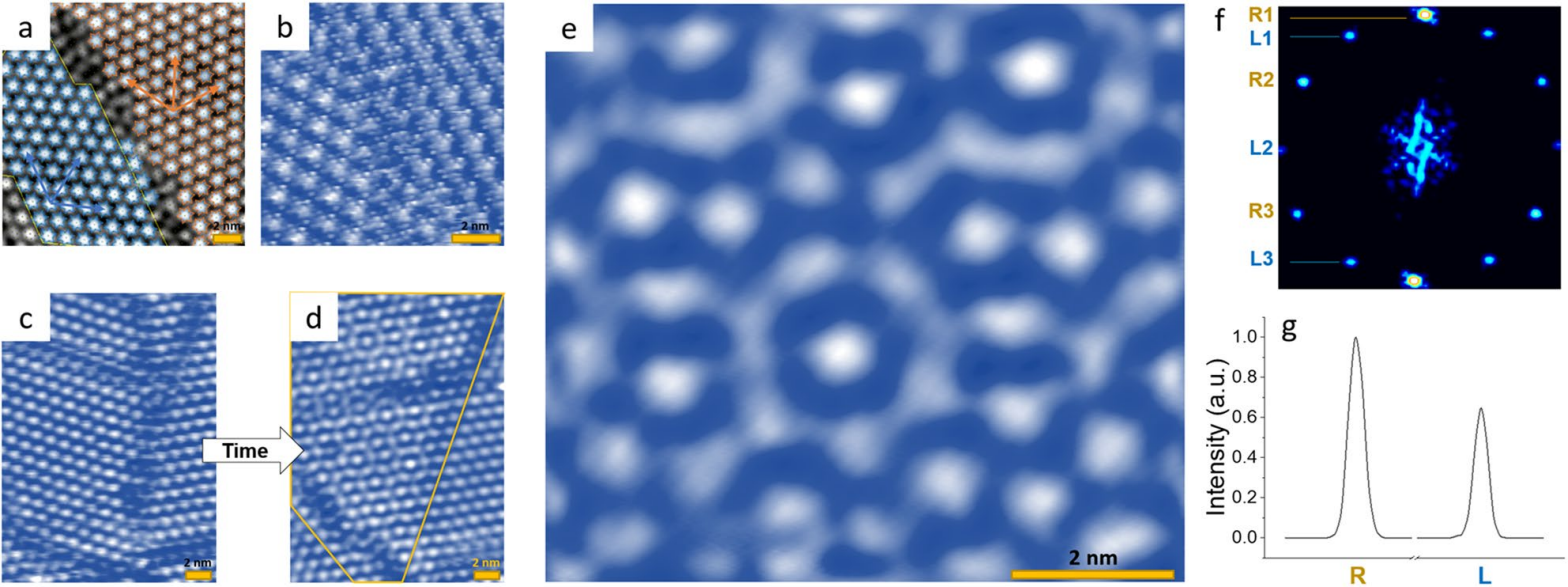
STM images of mirrored and interfering domains. (**a**) The photoinduced state with domains of L and R chirality (V = −0.8 V, I = 1 nA). An atomic lattice fit was used to accurately locate the domains and the David stars of different colors were drawn to mark the L and R superlattices. (**b**) An atomically resolved domain wall between domains with different chiralities. (**c,d**) The transformation of the CDW multiple-chirality domain structure to domains of interfering CDWs. The images are taken at 10-min intervals. (**e**) an enlarged image of the CDW interference patterns (V = −0.8 V, I = 0.4 nA). The scale bar is 2 nm in each case. (**f**) A FFT of the section bounded by the yellow line shown in (**d**). (**g**) The total intensities in the L and R FFT peaks after correction of scanning artefacts.

To induce the formation of mirrored domains, we perform a laser quench using a single or multiple 50 fs near-infrared (800 nm) laser pulses to drive the sample inside the STM chamber out of equilibrium (Fig. 1d). After destroying the CDW order, the system spontaneously reverts back towards equilibrium, initially forming L and R domains in equal probability until symmetry is spontaneously broken, resulting thereafter in the prevalence of one type of domain. Here we investigate metastable states which form in this process, whose lifetime is sufficiently long to be observable by STM. (More details on the photoexcitation are given in the “Methods” section). The illuminated area ($\sim 200\times 100$ µm spot size) is large compared to the microscopic structures under investigation with the STM, so the illumination of the observed surface can be considered to be uniform. The sample is mounted on a cold finger in contact with either the liquid helium ($\sim 4$ K) or liquid nitrogen ($\sim 77$ K) bath. In both cases, the photoinduced domain states are stable on a timescale of minutes, but at 77 K significantly more relaxation is observed^24^. The switching laser fluence of about 3–5 mJ/cm^2^ is sufficient to form a number of such domains, which are sometimes intermixed with regions of an amorphous electronic phase^24,27^. The mirrored domains appear either alone, or in groups. Unfortunately, it is hard to estimate their density from present STM experiments, because relatively small areas are probed. A typical boundary between two different domains is shown in a large area image in Fig. 2a, while an atomically resolved image of a similar domain wall is shown in Fig. 2b. While the domain walls between domains of the same in-plane chirality usually tend to be straight with occasional kinks, as seen with STM^20–22,28^ in large area scans, and—as we shall see later—also in model simulations^1,29^, the domain walls between L and R domains tend to be ragged, and their width is not easily defined. We have found that similar, mirrored domains can also be induced by a current pulse from the STM tip, but this method of excitation is not very uniform, and the result is local and not very reproducible.

Apart from neighboring L and R-handed domains, we also observe domains containing characteristic ‘flower-shaped’ interference patterns (Fig. 2d, e), which appear both at 4 K as well as 77 K. Compared with single-chirality domains, such interference patterns are quite rarely observed. The observed regions were typically $\sim 10\times 10$ nm in lateral size and may be bounded by domain walls. Remarkably, the modulation amplitude of the LDOS (i.e., image contrast) in such patterns is approximately the same as for single L -or R-oriented domains. A fast Fourier transform (FFT) analysis of the interference region (of Fig. 2d) is shown in Fig. 2f. After eliminating scanning artefacts, an analysis of the intensities shows that the total intensity of the L and R components shown in Fig. 2f have intensities with ratio $\sim 2/3$. (The detailed analysis is given in the Supplementary Information). Distortions of shape may arise at the edges of such domains, due to the presence of DWs, and defects or imperfections. The interfering domains can also appear in the process of relaxation (see example in Fig. 2c, d). The interference patterns are observed to form spontaneously, and/or decay into L or R order on a timescale of minutes or less, which prevents more detailed investigations of electronic structure by scanning tunnelling spectroscopy.

Given that the observed LDOS pattern is an interference of L and R CDWs, an interesting question is how the S state modulation arises: is the LDOS modulation predominantly within the first layer, whereby the second layer possibly plays a subtle role, or are we observing a new state with two coupled fully hybridized layers? The role of the second layer is clarified by two additional steady-state experiments: (i) In equilibrium samples without domains (or domain walls) the S pattern is conspicuously absent when the first and second layers have opposite chirality. (The chirality of the second layer is observed by direct STM imaging through small purpose-made holes in the top layer—see Supplementary Information for details). (ii) An S state is observed after excitation of the selenide compound, 4Hb-TaSe_2_. Here the top layer has the same 1T polytype structure as 1T-TaS_2_, but the second layer has a 1H polytype structure, so its S pattern cannot be the result of interference between the first two layers. (see Supplementary Information for details). Altogether, the evidence clearly implies that the S pattern is primarily the result of CDW interference within the top layer.

A number of factors can influence the ratio of amplitudes of the L and R components in the S pattern within the top layer. In particular, the in-plane boundary conditions surrounding a domain are likely to play an important role in determining the phase and amplitude of each of the two CDW components within the domain. The stability of the domain structure itself is topologically protected^22^, so the domain walls appear as an externally defined texture which imposes lateral boundary conditions for the CDW formation within the domain. We note that the domain structure itself is not independent of interlayer CDW stacking^25,30^, so the S pattern *is* indirectly linked with the inter-layer stacking. Indeed, recent calculations show that an underlying periodic potential from the layer below plays a very important role in determining the orientation of quasiparticle interference patterns in confined 1T-TaS_2_ nanostructures^31^. On the other hand, the absence of an S pattern in a sample without domains confirms that in-plane domain walls are essential for the S state to be observed.

In metastable states which contain domains (irrespective of how they are created), the domain walls of the second layer are faintly discernible, yet the CDW pattern within the top layer domains does not show any significant change of the LDOS modulation due to the phase-shifted layer underneath^20^. In case of the mosaic or hidden state, the domain walls in different layers are never reported to be in register directly above each other, which is consistent with the fact that DWs repel each other^1,29^. Finally, if the observed interference was a purely interlayer effect, it would very likely be seen every time we see a domain that is surrounded by domains of different chirality. An exception would occur if the domain in the top layer was in perfect register with a domain of the same size and orientation in the layer below, which is highly unlikely because of Coulomb repulsion. We will discuss the significance of this further in the Discussion.

## Emergent domain structures within a correlated electron model

**Figure 3:**
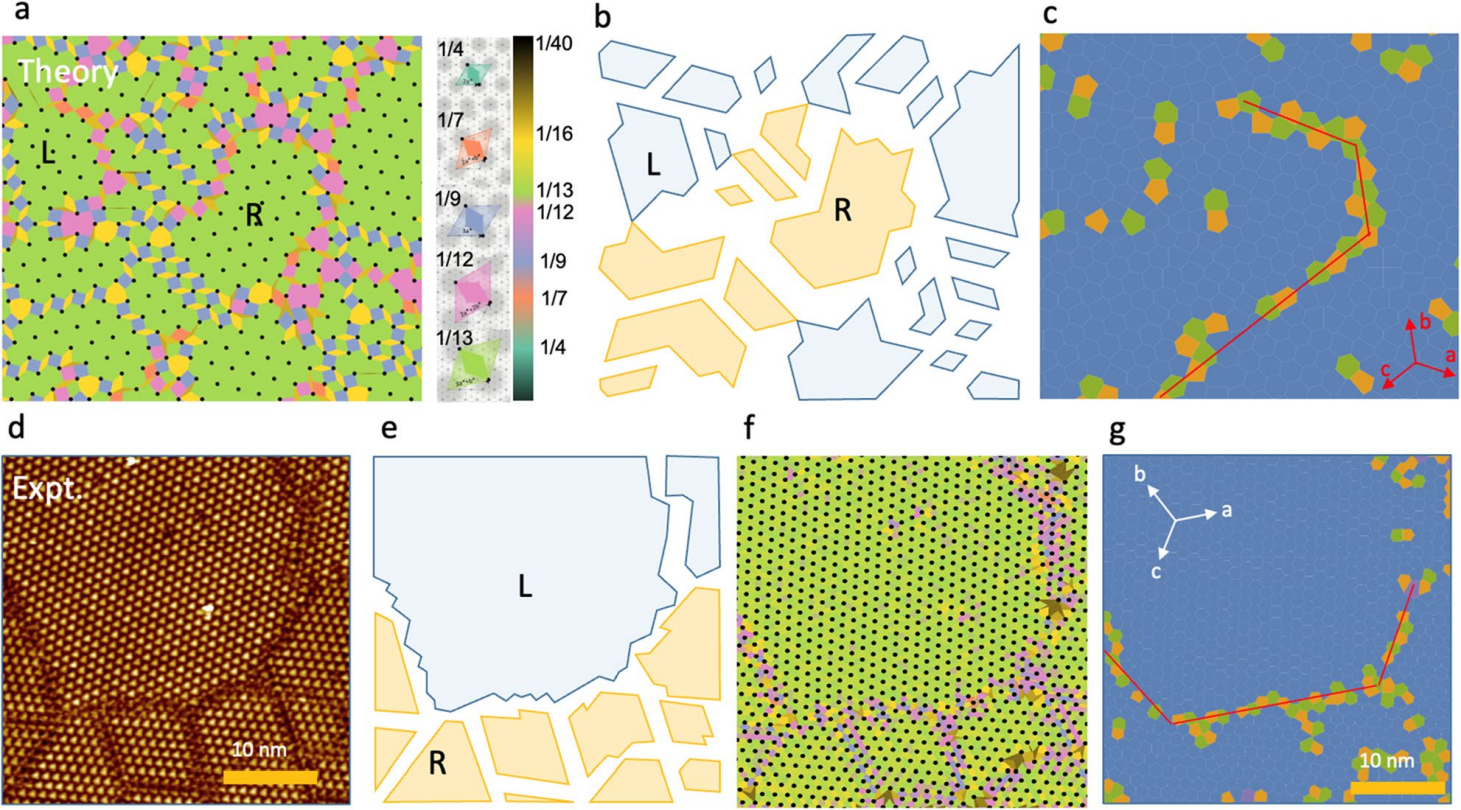
Comparison of the Monte Carlo model of the photoinduced state (**a–c**) with the STM measurements (V_tip_ = −0.8 V, I = 1 nA) (**d–g**). (**a**) Monte Carlo model calculation result, where the distances between neighboring polarons are shown with different colors. (**b**) A schematic showing the L and R domains. (**c**) The same data represented in the form of a Voronoi lattice, where yellow, blue and green represent n = 5, 6, and 7-sided n-gons respectively where n is the number of sides of an n-gon. (**d–g**) Experimental images: (**d**) shows an original STM image, while (**e–g**) show the experimental data using the same representation as for the MC calculation in the first row. The red lines in (**c,g**) indicate the atomic lattice vector directions.

In order to obtain insight into ordering of the mirrored domains, we first compare the measurements to the results of Monte-Carlo calculations of sparsely filled, screened, correlated electrons within a charge lattice gas (CLG) model on a hexagonal lattice^1^ in the Wigner crystal limit. The use of this model is justified by its success in describing the phase diagram of the material, particularly the commensurate phases at different fillings, the domain structure, and particularly, the presence of an amorphous phase^1,21,22,24^. The filling $f$ = 1/13 for the $\sqrt{13}\times \sqrt{13}$ superlattice in the C state. We start the simulation with polarons at random positions and perform Monte Carlo simulations of their discrete movements while lowering the temperature, eventually locking them in the lowest energy configuration at zero temperature. During cooling, ordered domains start appearing on the underlying atomic lattice. For a filling of exactly 1/13 they eventually merge to a single domain, while for fillings that slightly deviate from this value, we obtain multiple domain states^1^. Figure 3a shows an example of the simulated end-state with both domain orientations present. The black dots in Fig. 3a represent the polaron positions. Initially (at short times in the MC simulation), L and R domains appear in equal proportion, as there is no external symmetry breaking in the model (Fig. 3b). For very short annealing times, the crystal is frozen in the multi-chirality configuration. With increasing MC annealing time, the probability of finding mirrored domains in the simulation drops, showing that the presence of the mirrored domains is energetically more costly due to the relatively thick and ragged domain walls (see the Supplementary Information). The model calculation results can be directly compared to the experimental image shown in Fig. 3d–f.

Our experiments show that domains with different chiralities can be reached through processes in which the relaxation path goes through an intermediate state with restored mirror symmetry (Fig. 2c, d). When relaxing back to the $\sqrt{13}\times \sqrt{13}$ commensurate CDW state, the charge density wave may choose to form a domain with a chirality, that is not necessarily the same as the chirality of the surrounding CDW. This relaxation path may be aided by the high energy cost of L/R domain walls in the mixed state, as suggested by the MC calculations.

Colored tiling performed with diamond shapes (see legend in Fig. 3a), connecting neighboring polarons highlights the deviations from the $\sqrt{13}\times \sqrt{13}$ C structure (Fig. 3c)^1,27^. The most commonly observed color (light green) corresponds to the superlattice unit cell of the commensurate CDW. In the model, the domains are homogeneously green, while in the experimental image occasional deviations are seen due to measurement errors. In the domain walls the polarons can be either more closely or more sparsely packed, which is evident from the coloring.

To examine more closely the ordering within the domain walls we determine the number of nearest neighbors of each polaron by constructing a Voronoi diagram (Fig. 3c, g). Within domains, the polarons form a hexagonal superlattice, where each polaron has six nearest neighbors and its Voronoi cell is a hexagon. Adding straight domain walls between domains with the same chirality is effectively the same as stretching the lattice in one direction, which linearly deforms the hexagonal Voronoi cells. In this case, the number of nearest neighbors for each particle is conserved. On the other hand, when we look at the domain wall between two domains with different superlattice directions, we cannot simply represent it as a linear deformation of the lattice. Between L and R domains, additional particles are needed between domains, which manifests itself as a combination of pentagons and heptagons in the Voronoi diagram. In Fig. 3c, g, we show the Voronoi diagram of the model and a similar experimental image respectively. We color the n-gons yellow, blue and green for n = 5, 6 and 7 respectively. Both of the images are predominantly blue (covered by hexagons), with the large majority of the hexagons coming from within domains. Domain walls between domains of the same type are also predominantly formed of hexagons, where pentagons and heptagons still appear at the domain wall crossings (which cannot be represented simply as a linear deformation of the lattice). In domains with different superlattice directions, the domain walls are predominantly formed of non-hexagons. For clarity, the chirality of the domains is highlighted in Fig. 3b, e with semi-transparent blue and orange shapes, where we can clearly see that pentagons and heptagons clearly highlight the boundaries between the mirroring domains. We note that this is somewhat less clear in the experimental image, due to the errors in fitting the polaron positions. The heptagon can be explained as a consequence of an additional infinite 60° wedge of material squeezed into the lattice and the pentagon as a removal of a 60° wedge^32^, both of which thus deform the lattice. A pentagon–heptagon (H–P) pair therefore adds a wedge and removes another wedge, leaving the material with an additional semi-infinite stripe of unit cells^32^. H–P pairs in the domain wall crossings between domains of the same type thus add a nonzero-thickness stripe of material, which is the domain wall itself. A H–P pair is topologically equivalent to a crystal dislocation on a hexagonal lattice. In this sense, both free standing pentagons and heptagons, as well as their pairs are topologically protected. On the other hand, the domain walls between domains of different directions are composed of a series of such H–P pairs. In analogy to graphene, a series of such pairs leads to a nonzero angle between the neighboring lattices^32^, which is equivalent to the case that we discuss here. Additionally, since the H–P pairs highlight the domain walls very well, we can use them as a guide to determine the domain wall directions. In Fig. 3c, g, we draw additional red lines that are aligned with the lattice direction as a guide to the eye. We can see that the mirror domain walls roughly align with the lattice direction. A further detailed discussion about domain wall directions can be found in the Supplementary Information.

## Discussion

While the mixed L/R domain state can be described by the CLG model quite well, to describe the observed interference, we need to go beyond the strongly correlated classical polaron model. While Fermi surface (FS) nesting is considered to be rarely responsible for CDW formation in 2-dimensional materials, 1T-TaS_2_ FS nesting was suggested to contribute to the CDW instability^16^. Irrespective of the extent of such nesting, we may consider a picture of the CDW, in which FS electrons form interference patterns of L and R-handed superstructures. The H state is metallic, and the system has a Fermi surface, so we may discuss the system behavior in terms of the quasiparticle picture. Starting from a Schrödinger equation description of the CDW^14^, with degenerate L and R solutions for the broken symmetry ground state, a linear combination of states describes the superposition. This is true irrespective of the detailed interaction Hamiltonian, but requires the assumption that the state can be described by a Schrödinger Hamiltonian^14^. The L and R CDWs can thus be described in terms of the sum of one-directional CDWs:${A}^{m}\left(\stackrel{\rightharpoonup}{r}\right)=Re\left({\sum }_{d}{e}^{i\stackrel{\rightharpoonup}{{k}_{d}^{m}}\stackrel{\rightharpoonup}{r}}\right)$, where the sum over $d\in \{\mathrm{1,2},3\}$ represents the three symmetry directions of the L and R CDWs. $m\in \{L,R,0\}$ is the wavevector index, representing the R and L superlattices, and the atomic lattice respectively. $\stackrel{\rightharpoonup}{{k}_{d}^{m}}$ are the three reciprocal in-plane wavevectors for the two possible chiralities of the superlattice, $\stackrel{\rightharpoonup}{{k}_{d}^{L}}$ and $\stackrel{\rightharpoonup}{{k}_{d}^{R}}$ are the wavevectors for the positive and negative rotation angle of the superlattice with respect to the atomic lattice, and $\stackrel{\rightharpoonup}{{k}_{d}^{0}}$ is the atomic lattice wavevector.

**Figure 4:**
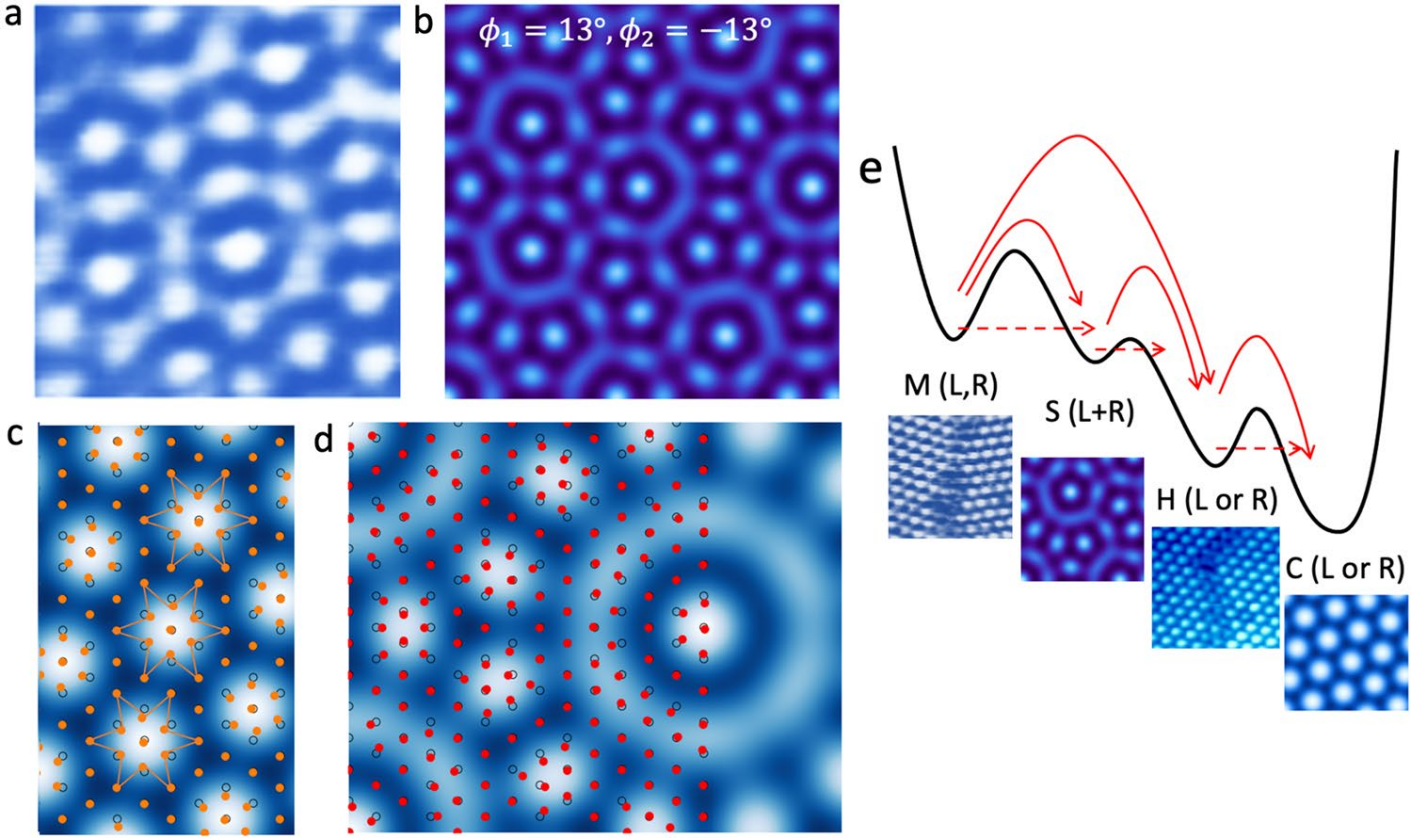
Modelling of the CDW interferences and lattice displacements. (**a**) An experimental STM interference pattern (V_tip_ = −0.8 V, 0.4 nA). (**b**) The patterns obtained as the superposition of L and R-handed CDWs with $\pm 13^\circ$ L and R CDWs. (**c**) The atomic displacements for one chirality within the simple tight-binding model. (**d**) Monte Carlo simulations of the movement of atoms (colored dots) from their initial positions (black circles) with a double CDW potential with $\pm$ 13.9$^\circ$, corresponding to ideal L and R commensurate structures of the $\sqrt{13}\times \sqrt{13}$ superlattice. (**e**) A free energy diagram showing the relaxation of the mixed state (M) via the superposition state (S) to the hidden (H) state and eventually to the single-domain C state. Relaxation can proceed by various thermally activated processes (solid arrows) or tunneling (dashed arrows).

The observed interference of the L and R CDWs (Fig. 4a) is described by ${A}^{int}\left(\stackrel{\rightharpoonup}{r}\right)=a{A}^{L}\left(\stackrel{\rightharpoonup}{r}\right)+b{A}^{R}\left(\stackrel{\rightharpoonup}{r}\right)$, with L and R amplitudes $a$ = $b$ is shown in Fig. 4b. We show the single chirality and double superlattices superimposed on the atomic lattice in Fig. 4c, d respectively. The calculated ${A}^{int}\left(\stackrel{\rightharpoonup}{r}\right)$ matches very well to the STM measurements for ${\theta }_{L,R}\pm 13^\circ$. Note that this angle is not equal to the geometric $\sqrt{13}\times \sqrt{13}$ superlattice angle ${\theta }_{S}=13.9^\circ$, where the pattern is different (see Fig. 4d) (interference plots for different ${\theta }_{L,R}$ are shown in the Supplementary Information). This discrepancy of $\sim 0.9^\circ$ between ${\theta }_{S}$ and ${\theta }_{L,R}$ is quite significant, and implies that the system is frustrated with respect to the ideal superlattice structure. We may attribute this frustration to the fact that the FS nesting wavevectors ${\theta }_{N}$ are not aligned with ${\theta }_{L,R}$^16^: In the high-temperature IC state, the CDW is aligned with the lattice (${\theta }_{IC}=0$), which is approximately aligned with the FS nesting wavevectors. In the NC phase, the CDW rotates to ${\theta }_{NC}\simeq 12\pm 1^\circ$, and eventually to $13.9^\circ$ in the C phase. In the superposition (S) phase, the CDW angles ${\theta }_{L,R}$ are thus in between the C phase and the NC phase.

It is interesting to consider the role of the atomic displacements in the superposition state. In this material, a Kohn anomaly is observed, associated with the IC transition, indicating that *e-p* coupling is strong near the IC wavevector 0.284 ${{\varvec{q}}}_{{\varvec{a}}}$, where ${{\varvec{q}}}_{a}=2\pi /{\varvec{a}}$ is the reciprocal lattice vector along the ***a*** crystal lattice direction^33,34^, implying that the periodic lattice displacements are closely following the electronic order. We can apply a simple tight binding model in order to consider the atomic displacements in response to the charge density in the superposition state (see SuppIementary Information for details of the calculation). The predicted single chirality displacements are shown in Fig. 4c. Note that the displacements of the outer 6 atoms are in neutral positions, and are not displaced, which is not the commonly presented description of the polaronic lattice deformations (Fig. 1b). For the interference of L and R CDWs, the resulting atomic displacements correspond to the vector sum of the displacements arising from the L and R CDWs, as shown in Fig. 4d. Unfortunately, considering the state is metastable, and is mesoscopically embedded within the mixed chirality domains, the atomic displacements may be difficult to measure experimentally.

A conceptual free energy diagram is shown in Fig. 4e, indicating the cascade of transitions from the mixed state (M), with L and R domains spatially separated by domain walls, to the L + R superposition state (S) which decays to the H state (either L or R), and eventually to a L or R commensurate ground state (C). The barrier energy separating these states is primarily determined by the non-equilibrium local boundary conditions, imposed by the domain walls. The periodic potential of both the lattice, and CDW ordering in the layer below is also likely to influence the stacking energy. It is difficult to define a single order parameter for the overall free energy diagram, and the free energy should be understood to be a multidimensional, time-varying order-parameter landscape, which depends on the instantaneous domain configuration. Unfortunately, this cannot be easily represented by a single- or two-dimensional dimensional plot, but one may simply imagine that a different order parameter is relevant for each of the transitions represented in the single-dimensional free energy diagram shown in Fig. 4e, and the free energy curve changes as the domain structure evolves with time. A more elaborate band structure calculation of the interfering CDW phase may help in revealing the origin of its metastability for particular cases, particularly when the interactions between TaS_2_ layers are considered.

It is worth discussing the significance of the presently reported *intra*-layer interference in the context of commonly observed multi-layer interferences (Moiré patterns), such as previously observed on the surface layer of Nb-substituted 1T-TaSe_2_ by Ohta et al.^8^. In the latter case the observed modulation arises from the interaction of the two topmost layers with different 2D chiralities. In the present case, we have argued in the results section that the layer below is not directly responsible for the L + R interference, and is fundamentally different from such inter-layer Moire patterns. The observation of an intra-layer interference appears as a manifestation of the quantum behavior of the CDW for electrons confined within a domain created during the quench process. We note that a related effect of intra-layer quantum interference arising from in-plane electron confinement within triangular heterostructures was recently reported, although only irregular interference patterns were observed^28^. In principle, the L + R interference effect within domains is possible for all CDW superlattices that have broken in-plane mirror symmetry with respect to the crystal lattice (e.g.$\sqrt{7}\times \sqrt{7}$ or $\sqrt{21}\times \sqrt{21}$)^1^, but to our knowledge, this is the first time it has been observed experimentally.

## Conclusions

We conclude that transient non-trivial metastable CDW interferences and novel orders that are not known under equilibrium conditions can be observed under non-equilibrium conditions. The new metastable interference state—irrespective of whether it is described in terms of a correlated electron model, a periodic lattice distortion arising from electron–phonon coupling, or a superposition of Fermi electrons with wavevectors of opposite chirality—highlights the wave/particle duality of metastable systems close to localization. Within the correlated electron model, the mixed L/R phase is clearly seen as a natural consequence of the non-equilibrium nature of the system. A density functional theory calculation might further reveal the origin of the metastability of the observed states on the microscopic level^28^, but the boundary conditions imposed by domain walls that are deemed essential for an accurate representation of the observed superstructures, impose unrealistic demands on such calculations at present. While full inter-layer hybridization is apparently ruled out on experimental grounds, inter-layer interactions might aid in stabilizing the superposition state, here observed experimentally as a transient state for the first time. Finally, we note that boundary conditions are clearly an important factor that can be manipulated in order to create new states of matter on the mesoscopic scale under non-equilibrium conditions.

## Methods

### Sample preparation

We grow the 1*T*–TaS_2_ crystals by chemical transport method using iodine as a transport agent. The average dimensions of the samples are $2\times 2\times 0.1$ mm. The samples are glued to the STM sample holder with UHV-compatible silver paste and cleaved under UHV.

### STM measurements and optical switching

For the STM measurements we use a UHV LT Nanoprobe (Scienta Omicron) with optical access through obliquely angled side windows. For the photoexcitation, we use a 100 kHz, 800 nm laser system, with 50 fs pulses. The number of pulses was selected using an acousto-optic modulator. The laser beam is guided into the STM chamber using an automatic stabilizing system with a positioning precision of $<5$ µm. The laser beam profile was carefully determined externally with a CCD camera. A scanning electron microscope mounted above the STM allowed for precise tip positioning. The center position of the laser beam was determined from the perimeter defined by the border between the equilibrium and switched states. The peak fluence on the sample is kept between 3 and 5 mJ/cm^2^. The tip voltage was adjusted for maximum spatial modulation of the LDOS (typically −0.8 V w.r.t. the sample) enabling fast scanning of metastable domains. The tip currents were kept below 1 nA to avoid influencing the dynamics as much as possible. The usual time between the optical excitation and the STM scanning varies between minutes to hours, mainly depending on the time needed to find a suitable area that exhibits interferences.

### Charge lattice gas model

The model of classical chiral domains is based on the charged lattice gas (CLG) Hamiltonian $H={\sum }_{i,j}V\left(i,j\right){n}_{i}{n}_{j}$ where ${n}_{i}$ is the occupational number of a polaron at site $i$ with values either 0 or 1 and $V(i,j)={V}_{0}exp(-{r}_{ij}/{r}_{s})/{r}_{ij}$ is the Yukawa potential that describes the screening. ${V}_{0}={e}^{2}/{\epsilon }_{0}a$ in CGS units and ${r}_{ij}=|{r}_{i}-{r}_{j}|$, where ${r}_{i}$ is the dimensionless position of the $i$-th polaron and ${r}_{s}$ is the dimensionless screening radius. The dimensions are normalized, so that the value 1 for both $|{r}_{i}|$ and ${r}_{s}$ corresponds to one lattice constant $a$. The value of ${r}_{s}\to \infty$ (see Ref.^27^ for details) in the Wigner crystal limit. $e$ and ${\epsilon }_{0}$ are the electron charge and static dielectric constant of the material respectively. Polarons can only occupy the sites of the underlying triangular lattice. The ratio of polarons in the system divided by the total number of lattice sites is expressed as the filling $f$. The Monte-Carlo method used to simulate the model was described previously^1^, where we studied the phase diagram of such a system at fixed values of $f$ and optimized ${r}_{s}$. The CLG interpretation of polarons assumes a system of interacting phonons and repulsive electrons that is canonically transformed into a system of interacting small polarons in the strong electron–phonon coupling limit. We neglect spin effects, assume a screened Coulomb interaction, and assume that the hopping of polarons $\widetilde{t}\ll {V}_{0}$, which is justified by the static nature of observed charges and set to zero in this model.

### Periodic lattice distortion model

We use the Lennard–Jones equation $V\left(r\right)=4\varepsilon \left[{\left(\frac{\sigma }{r}\right)}^{12}-{\left(\frac{\sigma }{r}\right)}^{6}\right]$ to model the potential energy between the atoms. To normalize the model, we set $\sigma ={2}^{-\frac{1}{6}}$ so that the neighboring two particles are in equilibrium, when they are at the distance of $r=1$ and $\varepsilon =1$ so the depth of the potential is ${V}_{min}=-1$. We introduce the CDW as an outside potential energy in form of the cosine wave with the depth of $U$. The particles are positioned on a perfect hexagonal lattice and their movement is simulated using a Monte-Carlo simulation. The calculations give *qualitatively* the same result for $U$ ranging from 0.1 to > 100.

### Supplementary Information

Supplementary Information.

## Data Availability

All of the data supporting the conclusions are available within the article and the Supplementary Information. Additional data are available from the corresponding author upon reasonable request.

## References

[1] Vodeb J, Kabanov VV, Gerasimenko YA, Ravnik J, van Midden MA, Zupanic E, Sutar P, Mihailovic D (2019). Configurational electronic states in layered transition metal dichalcogenides. New J. Phys..

[2] Coleman R, Giambattista B, Hansma P, Johnson A, McNairy W, Slough C (1988). Scanning tunnelling microscopy of charge-density waves in transition metal chalcogenides. Adv. Phys..

[3] Hall J, Ehlen N, Berges J, van Loon E, van Efferen C, Murray C, Rosner M, Li J, Senkovskiy BV, Hell M (2019). Environmental control of charge density wave order in monolayer 2H-TaS2. ACS Nano.

[4] McMillan W (1975). Landau theory of charge-density waves in transition-metal dichalcogenides. Phys. Rev. B.

[5] Villain J (1980). Commensurate-incommensurate transition of krypton monolayers on graphite: A low temperature theory. Surf. Sci..

[6] Bak P (1982). Commensurate phases, incommensurate phases and the devil's staircase. Rep. Prog. Phys..

[7] Wilson JA, Di Salvo F, Mahajan S (1975). Charge-density waves and superlattices in the metallic layered transition metal dichalcogenides. Adv. Phys..

[8] Li, W. & Naik, G.V. Reorganization of CDW stacking in 1T-TaS2 by an in-plane electrical bias. *APL Mater.* **9**, 18 (2021).

[9] Gao, J., Zhang, W., Si, J., Luo, X., Yan, J., Jiang, Z., Wang, W., Lv, H., Tong, P., Song, W. *et al*. Chiral charge density waves induced by Ti-doping in 1T-TaS2. *Appl. Phys. Lett.* **118**, 256 (2021).

[10] Liu, G., Qiu, T., He, K., Liu, Y., Lin, D., Ma, Z., Huang, Z., Tang, W., Xu, J., Watanabe, K. *et al*. Electrical switching of ferro-rotational order in nanometre-thick 1T-TaS2 crystals. *Nat. Nanotechnol*. **1**, 1–7 (2023). 10.1038/s41565-023-01403-5 37169899

[11] Ohta S, Kobayashi S, Nomura A, Sakata H (2021). Electronic state modulation of the Star of David lattice by stacking of 13 × 13 domains in 1T- TaSe2. Phys. Rev. B.

[12] Zong, A., Shen, X., Kogar, A., Ye, L., Marks, C., Chowdhury, D., Rohwer, T., Freelon, B., Weathersby, S., Li, R. et al. Ultrafast manipulation of mirror domain walls in a charge density wave. *Sci. Adv.* **4**, eaau5501 (2018). 10.1126/sciadv.aau5501 PMC6195337 30345365

[13] Bardeen J (1980). Tunneling theory of charge-density-wave depinning. Phys. Rev. Lett..

[14] Miller J, Wijesinghe A, Tang Z, Guloy A (2012). Correlated quantum transport of density wave electrons. Phys. Rev. Lett..

[15] Vodeb, J., Diego, M., Vaskivskyi, Y., Gerasimenko, Y., Kabanov, V. & Mihailovic, D. *Emergent False Vacuum Decay Processes in a Two-Dimensional Electronic Crystal: Experiment vs. Simulations on a Noisy Superconducting Quantum Processor*. arXiv Preprint arXiv:2103.07343 (2021).

[16] Sohrt C, Stange A, Bauer M, Rossnagel K (2014). How fast can a Peierls–Mott insulator be melted?. Faraday Discuss..

[17] Scruby C, Williams P, Parry G (1975). The role of charge density waves in structural transformations of 1T TaS2. Philos. Mag..

[18] Sipos, B., Kusmartseva, A. F., Akrap, A., Berger, H., Forró, L. & Tutivs, E. From Mott state to superconductivity in 1T-TaS 2. *Nat. Mater*. **7**, 960 (2008). 10.1038/nmat2318 18997775

[19] Stojchevska L, Vaskivskyi I, Mertelj T, Kusar P, Svetin D, Brazovskii S, Mihailovic D (2014). Ultrafast switching to a stable hidden quantum state in an electronic crystal. Science.

[20] Ma L, Ye C, Yu Y, Lu XF, Niu X, Kim S, Feng D, Tománek D, Son Y-W, Chen XH (2016). A metallic mosaic phase and the origin of Mott-insulating state in 1T-TaS 2. Nat. Commun..

[21] Cho D, Cheon S, Kim K-S, Lee S-H, Cho Y-H, Cheong S-W, Yeom HW (2016). Nanoscale manipulation of the Mott insulating state coupled to charge order in 1T-TaS 2. Nat. Commun..

[22] Gerasimenko YA, Karpov P, Vaskivskyi I, Brazovskii S, Mihailovic D (2019). Intertwined chiral charge orders and topological stabilization of the light-induced state of a prototypical transition metal dichalcogenide. Npj Quantum Mater..

[23] Ravnik J, Vaskivskyi I, Mertelj T, Mihailovic D (2018). Ultrafast jamming of electrons into an amorphous entangled state. Phys. Rev. B.

[24] Ravnik J, Diego M, Gerasimenko Y, Vaskivskyi Y, Vaskivskyi I, Mertelj T, Vodeb J, Mihailovic D (2021). A time-domain phase diagram of metastable states in a charge ordered quantum material. Nat. Commun..

[25] Stahl Q, Kusch M, Heinsch F, Garbarino G, Kretzschmar N, Hanff K, Rossnagel K, Geck J, Ritschel T (2020). Collapse of layer dimerization in the photo-induced hidden state of 1T-TaS 2. Nat. Commun..

[26] Le Guyader L, Chase T, Reid A, Li R, Svetin D, Shen X, Vecchione T, Wang X, Mihailovic D, Dürr H (2017). Stacking order dynamics in the quasi-two-dimensional dichalcogenide 1 T-TaS2 probed with MeV ultrafast electron diffraction. Struct. Dyn..

[27] Gerasimenko YA, Vaskivskyi I, Litskevich M, Ravnik J, Vodeb J, Diego M, Kabanov V, Mihailovic D (2019). Quantum jamming transition to a correlated electron glass in 1T-TaS 2. Nat. Mater..

[28] Park JW, Lee J, Yeom HW (2021). Zoology of domain walls in quasi-2D correlated charge density wave of 1T-TaS 2. Npj Quantum Mater..

[29] Karpov P, Brazovskii S (2018). Modeling of networks and globules of charged domain walls observed in pump and pulse induced states. Sci. Rep..

[30] Ritschel, T., Trinckauf, J., Koepernik, K., Büchner, B., Zimmermann, M.V., Berger, H., Joe, Y., Abbamonte, P. & Geck, J. Orbital textures and charge density waves in transition metal dichalcogenides. *Nat. Phys.* **11**, 328 (2015).

[31] Ravnik, J., Vaskivskyi, Y., Vodeb, J., Aupivc, P., Vaskivskyi, I., Golevz, D., Gerasimenko, Y., Kabanov, V. & Mihailovic, D. Quantum billiards with correlated electrons confined in triangular transition metal dichalcogenide monolayer nanostructures. *Nat. Commun.* **12**, 1 (2021). 10.1038/s41467-021-24073-0 PMC8213767 34145280

[32] Yazyev OV, Louie SG (2010). Topological defects in graphene: Dislocations and grain boundaries. Phys. Rev. B.

[33] Machida Y, Hanashima T, Ohkubo K, Yamawaki K, Tanaka M, Sasaki S (2004). Observation of soft phonon modes in 1 T-TaS2 by means of X-ray thermal diffuse scattering. J. Phys. Soc. Jpn..

[34] Ziebeck K, Dorner B, Stirling W, Schollhorn R (1977). Kohn anomaly in the 1T2 phase of TaS2. J. Phys. F Met. Phys..

