# Supplementary material for: Chiral domain dynamics and transient interferences of mirrored superlattices in nonequilibrium electronic crystals
Source: Sci Rep. 2023 Nov 10;13:19622. doi: 10.1038/s41598-023-46659-y (PMC10638312; doi:10.1038/s41598-023-46659-y)
Supplement: Supplementary file 1 — Supplementary Information. [file 41598_2023_46659_MOESM1_ESM.pdf]

Supporting information

Chiral domain dynamics and transient interferences of mirrored superlattices in nonequilibrium electronic crystals

J. Ravnik,[1,2] J. Vodeb[1], Ye. Vaskivskyi[1], M. Diego[1], R.Venturini[1], Ya. Gerasimenko[3], V. Kabanov[1], A. Kranjec[1], D. Mihailovic[1,3]

The measurement procedure

We glue a bulk 1*T*-TaS_2 sample to the STM holder and insert it into the STM. We cleave the sample in an ultrahigh vacuum to ensure a clean, non-contaminated surface. We use a UHV LT Nanoprobe (Scienta Omicron) device with optical windows, through which we introduce laser pulses for photoexcitation. We use a 100 kHz, 800 nm laser system, which produces 50 fs pulses. We use a single pulse or multiple pulses to photoexcite the sample, where the number of pulses ranges to as much as 10^6 pulses (corresponding to a 10-second exposure of the sample to the 10 kHz pulse train). The exposure time is controlled with an acousto-optic modulator. The effects of using a large number of pulses and higher fluences lead to many more interesting phenomena, which are out of the scope of this paper and are already well described in [1]. The laser spot size is very large ($\sim 200 \times 100$ μm) compared to the typical STM scan size (up to and rarely above $\sim 50 \times 50$ nm) and the microscopic structures under investigation. Due to the large difference in sizes (an STM scan is about 3 orders of magnitude smaller than the size of the beam), we can consider the observed surface to be uniformly excited, even though the beam has a Gaussian profile. The center position of the laser beam was determined from the perimeter defined by the border between the equilibrium and switched states and the local laser fluence at the point of measurement was recalculated given the distance from the center of the beam (for more details see reference [1]). We mostly see the mirrored domains at fluences of 3-5 mJ/cm^2, sometimes intermixed with regions of an amorphous electronic phase [1,2], but they do appear also at lower and higher fluences. The fluences between 3-5 mJ/cm^2 are a good choice, as they are high enough to create domain states, with a high possibility to observe domains of different chiralities, but not high enough to damage the sample.

The measurements were performed at either ~ 4 K or ~ 77 K (cooled by liquid helium or liquid nitrogen respectively). The lifetime of the photoinduced domain states at such temperatures is long enough to assume that the states are static and perform reliable measurements, regardless of whether we have only one orientation of domains or mirrored domains. In some cases, occasional changes in the domain structures are seen, which do not affect the measurement performance. Measuring a typical image takes between one minute and 10 minutes, while the domain structure usually stays largely unchanged on the timescale of hours or even days, thus changes in the domain structure are very rarely seen between two consecutive scans. At 77 K the domain relaxation is a bit more pronounced than at 4 K

and it mostly happens at the outskirts of the beam [1]. Conversely, we cannot claim this for the interfering domains. We have in most cases seen the interference pattern change (appear or disappear) between two consecutive scans (a few minutes) or even as an abrupt change during a scan, both at 77 K as well as at 4 K. The examples are described in detail in the following sections.

The tip voltage was adjusted for maximum spatial modulation of the LDOS (typically -0.8 V w.r.t. the sample) enabling fast scanning of metastable domains. The tip currents were kept below 1 nA to avoid influencing the dynamics as much as possible.

Scan of a large area, where domains of different chiralities are observed

Here we present a scan of a larger area with domains of different chiralities. Note that most of the domains are of one chirality, while there are three very large and a few smaller domains of the opposite chirality.

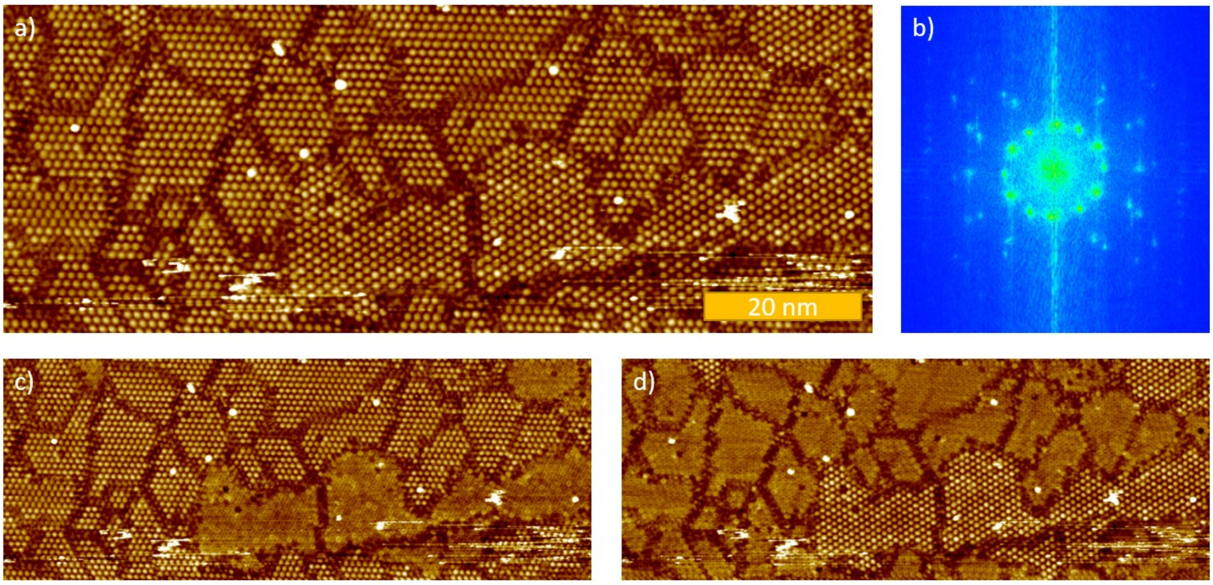

Figure S1. a) A larger area (110×40 nm), where domains of both chiralities were observed. b) Fourier transform of a). We can clearly see the 12 large main (inner) peaks (6 of them belong to each chirality). The outer, weaker peaks are higher-order peaks. c) and d) show the same as a), but with one chirality of the domains masked out, for easier interpretation of a).

Relaxation of domains

Both at 4 K and at 77 K we observe very little spontaneous relaxation of the photoinduced states on the timescale of STM measurements. In most cases, we see movements of individual atoms, but very rarely we see full domains collapse. Since the measurement itself perturbs the sample, a detailed analysis would be needed to estimate what portion of relaxation is caused by the STM tip and what

portion of the relaxation happens spontaneously. We have left the STM device scanning the same area for over six hours. We show the changes in Fig. S2.

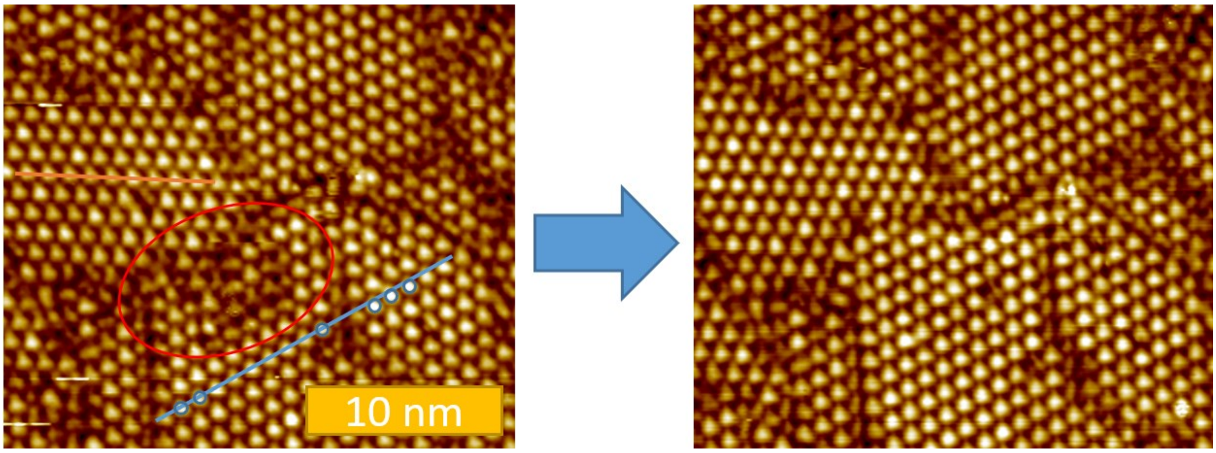

Figure S2. Relaxation of the domain over a period of six hours. We can see some rearrangement of the domains, but on a larger scale not much is changed. The red ellipse highlights the region with the most prominent changes. Blue and orange lines are guides to the eye for the orientation of the domains. Blue circles show the approximate positions of the peaks of CDW within the domains and aligning them to the blue line highlights the change of the CDW phase between the two domains of the same chirality.

Additionally, we have tried to induce relaxation by applying voltage pulses at the borders between the domains of different chiralities. The process strongly depends on the quality of the tip and is in cases of bad tip quality not very reproducible. The threshold voltages for inducing electronic changes in the sample and damaging the sample are sometimes very close and thus extreme caution is needed. Typically, we find that voltage pulses of about 1 V are enough to induce some changes to the electronic states. In Fig. S3 we show the process of relaxation of a chiral domain, using a series of 1 V pulses triggered at the approximately same position on the sample every time. In total, we have applied around ten voltage pulses, but we show only the two cases where an actual change was observed. Since the rearranging of the domains does not happen every time a pulse is applied, one has to be patient and not increase voltage too much, as this can lead to partial removal of the top layer(s) and the creation of holes in the sample.

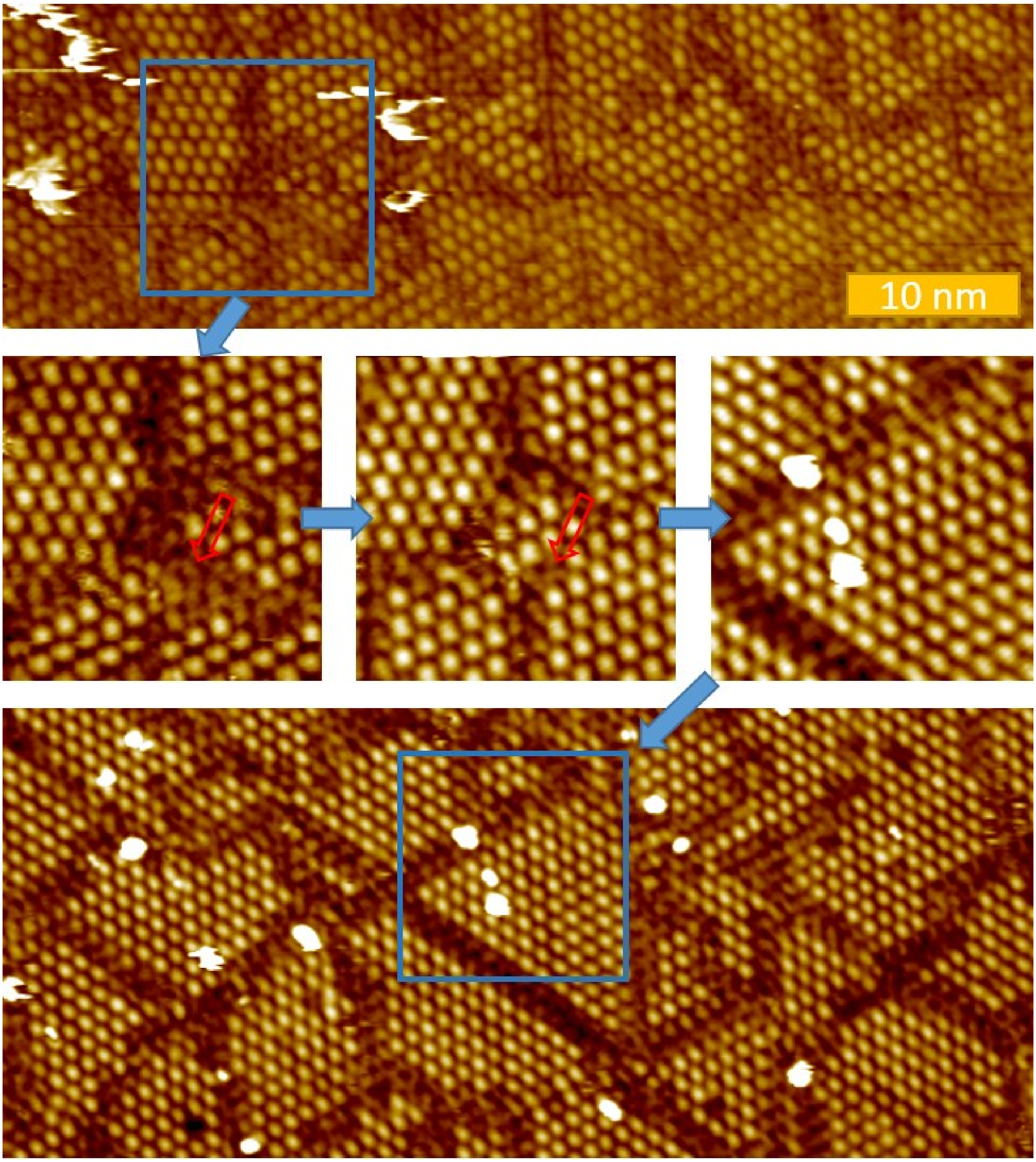

Figure S3. Relaxation of the domains after applying voltage pulses. Top: the larger area around the observed area before any pulses were applied, middle: the small observed area before and after applying voltage pulses, and bottom: extended area after the pulses were applied. The blue arrows are denoting the time sequence in which the images were taken, and the blue rectangles show the position of the small area within the larger area. The red arrows denote the approximate positions of the voltage pulses.

The detailed transition between the domains of different chirality through a domain with interfering chiralities

Here we show a relaxation process from the domains of different chiralities, through a domain with interfering chiralities to the domain state with only one chirality present in Fig. S4 a), b) and c). We

perform Fourier filtering on the domain with the chirality interference (filtered images in d) and f) are both taken from the image b)), which remarkably shows the remains of the domain structure that we observed before the transition and the hints of the domain structure after the domain structure. Thus the filtered data in d) resembles the domains in a) and the filtered data in f) resembles the domains in c). The time between the images is below 10 minutes for a) and b) and tens of minutes between b) and c).

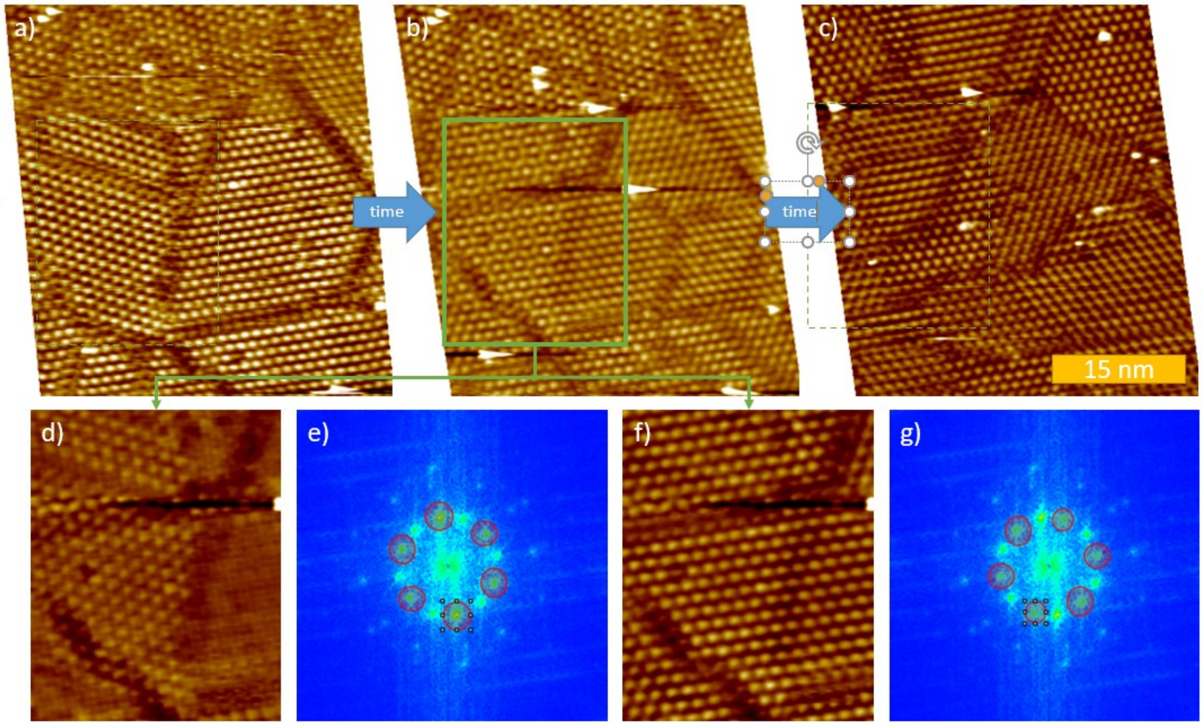

Figure S4. The relaxation of domains of different chiralities through a domain with interfering chiralities. a), b) and c) show approximately the same region at three different times. In a) we can see domains of different chiralities in the left and right parts of the image. In b), the chirality from the right side of the image propagates into the domain on the left, and we see a domain with the interfering chirality. c) shows the same region when the interfering domain relaxes to only one chirality. d) and f) show the zoom-in to the green rectangle from b) with an applied Fourier filter to only see the contribution of one of the chiralities. e) and f) show their respective Fourier transforms with filtered frequencies marked with red circles.

Fourier analysis of interfering chiralities

In order to perform the Fourier analysis, careful consideration of all the scanning data is required. For most of the data, only the Z-scans (height maps) are considered, since the STM is operating in the constant current mode. Taking into account the fact, that this mode highly depends on the sensitivity and speed of the feedback loop as well as on the tip movement speed, the real-life image which is obtained is in a quasi-constant-current mode. Taking into account both the height and current data for each of the STM scans, we can highly improve the analysis.

The 4 raw scans considered for the analysis are presented in Fig. S5. The top-row panels show Z(height)-scans up (**a** – forward/right, **e** – backward/left) and down (**i**, **m** respectively). The next row shows the I(current)-scans obtained as a result of low feedback speed. The last row represents 2D-FFTs of the corresponding scans (in each pair: left for H and right for I). All the data is presented as measured without any filtering or corrections.

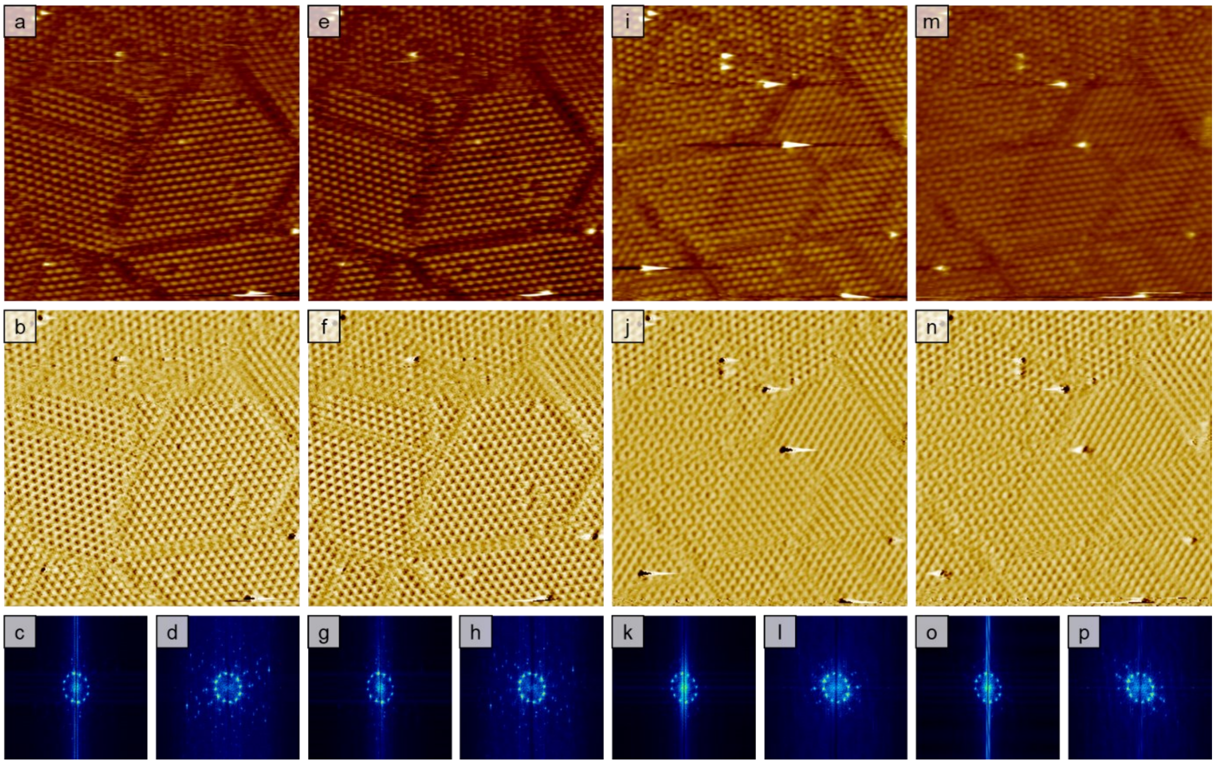

Figure S5. The original STM scans during which the interference was observed. a-d) up-forward. e-h) up-backwards. i-l) down-forward. m-p) down-backwards. Panels a, e, I, and m present Z (height) images in the constant-current (feedback) mode of STM. Panels b, f, j and n present I (current) images as a result of low feedback speed during the scans. Panels c, g, k, o present 2D-FFT for height and d, h, l, p – 2D-FFT for current images in the corresponding scans.

To examine the effect of feedback, the first scan in the series (Fig. S6a) before the interference appearance is considered. For the 2D-FFT calculation and quantitative analysis of both L and R chiralities, the same number of polarons in a single group with a similar symmetry is considered (marked in green areas). Figs. S6b,c show the 2D-FFTs for the height and current map respectively. As seen from the panels, there is a defined direction (vertical), along which the intensity of the FFT peaks is enhanced/suppressed. The same effect is easily seen in the bottom row of Fig. S5 – this is the result of the line-rastering noise.

Panel **d** of Fig. S6 presents the cross-sections of each FFT peak. Their peak values are presented in panel **e** together with the sum of Z and I FFT peaks. As can be seen in the figure, the total sensitivity of

the tip during the scan is direction-dependent but does not have any sharp features which are presented in Z-only or I-only data. This can be considered as the effect of tip shape. Fig. S6f shows the comparison of the FFT analysis performed for scans forward (Fig. S5a,b) and backward (Fig. S5e,f). Since the data is similar, our data can be considered regardless of the scan direction along the fast (X) axis.

Fig. S6g marks 4 different areas with R-only chirality of polarons on the scan down (Fig. S5m,n). Area 4 contains the other 3 areas. It can be considered the most precise since it covers the largest part of the original scan. Fig. S6h represents the results of FFT analysis for the areas marked in panel g. The reason for the different behaviour of area (3) is unknown but can be connected to bad statistics in different directions because of highly-elongated AOI.

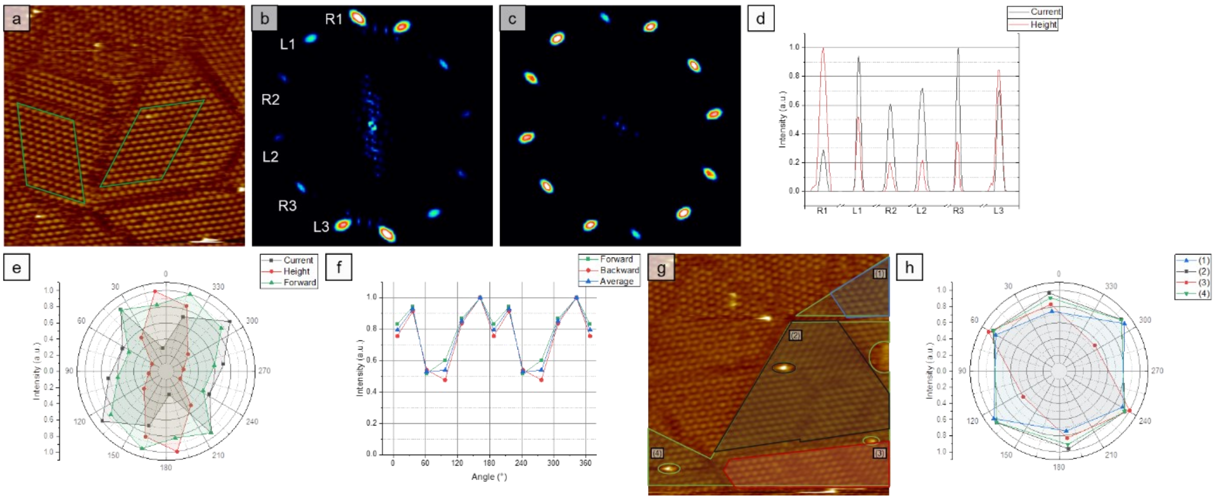

Figure S6. a) Z-scan of the sample before interference appears as in Fig. S5a with marked areas for quantitative 2D-FFT analysis containing the same amount of polarons with the same symmetry. b) 2D-FFT of areas marked in panel a with peaks labelled. c) 2D-FFT of the same areas in the I-scan as in Fig. S5b. d) Quantitative comparison of R and L FFT peaks in both Z and I-scans. e) Quantitative analysis of R and L FFT peaks considering the spatial directions. The green plot represents the sum of Z and I-scans. f) Comparison of FFT analysis for scans forward (Fig. S5a,b) and backward (Fig. S5e,f) and their average. g) Z-scan of the area with interference observed as in Fig. S5m with different parts of R-only chirality marked for analysis. h) Comparison of FFT analysis for areas marked in panel g. Both Z and I-scans are considered in each curve.

Fig. S7a,b show the interference area considered in the analysis as well as the R-only chirality for comparison (the same as Fig. S6g,h (4)) on height and current-maps respectively. The corresponding 2D-FFTs are shown in Fig. S7c,d. As can be seen from the FFT analysis (Fig. S7e), the intensity of all the L peaks is lower in comparison to all the R peaks. Also, peak R3 has the same feature, as one in Fig. S6h(4) which is almost twice less intense as compared to the other two R-peaks, marking elliptical asymmetry of the CDW intensity observed in this area.

One can perform an ellipse-to-circle correction on the resulted interference curve as shown in Fig. S7f (purple) to the peaks L2, R3 and L3 if considering the following axes of the ellipse: $a = average(R1, R2)$; $b = R3$, as well as the radius of circle $r = a$.

If we consider the total part of L chirality as $L = L1 + L2 + L3$ and R chirality as $R = R1 + R2 + R3$, for the FFT peaks with radial cross-sections as shown in Fig. SY3g, the result of $L \approx 0.648\ R$ is obtained. Fig. S7h shows the sum of cross-sections from panel g.

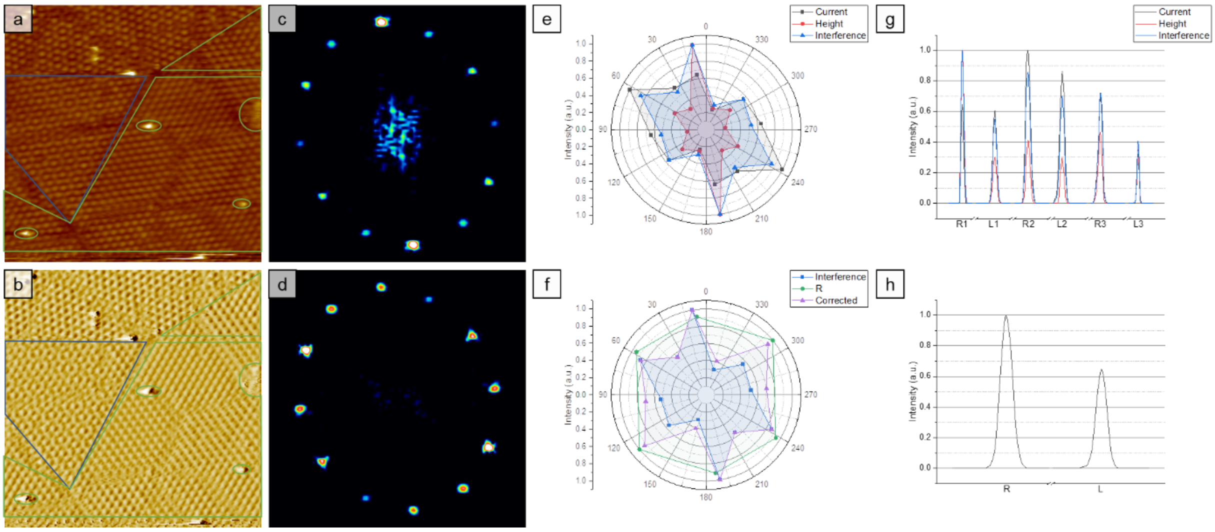

Figure S7. a,b) Z and I-scans with the interference area marked as well as R-only chirality. Both panels as in Fig. SY1m,n. c,d) 2D-FFT for the interference area in Z and I-scans. e) FFT analysis of the interference image. f) Comparison of the interference FFT with the FFT of R-only chirality. The corrected curve presents an ellipse-to-circle correction performed on the interference plot with R3 (129.6 ° and 309.6 °) peak corrected to an average value of R1 and R2. g) Radial cross-sections of each FFT peak for Z, I-scans and their sum. h) Sum of cross-sections for all R and L peaks after correction for the FFT peak-to-centre distance due to image drift during the scan. The peak of the L-curve is 64.8%.

One more piece of evidence of the similar intensities of the two chiralities in the interference pattern can be seen in the domain walls (DWs), visible in the STM scan. The area of interest marked in Fig. S7a,b. contains two DWs – a straight one corresponding to the R chirality and aligned to the R CDW in all other parts of the image (a “stronger” DW); and another straight DW which was observed before (Fig. S5a,e), aligned to the L chirality (a “weaker” DW). Considering the exact same position of this DW as on the previous scans, as well as the absence of any bottom-layer DW effects visible in other parts on the same scan at these conditions, we can attribute this “weaker” DW to the top layer the same way as the “stronger” one.

Appearance of the interfering CDW domain during a single scan

In one case, we were able to witness the switching to the domain with the interfering chiralities during a single STM scan. The scan was taken from the bottom to the top. As we can see, it starts with great atomic resolution of a single domain (it is unclear that this is a domain, since there are no visible domain walls, as the scanning area is too small and just covers a single domain). At approximately one third of the scan, the image changes abruptly: we lose the resolution and the pattern changes significantly. We do not know the exact cause, but a plausible explanation is that the single atom at the end of the tip either dropped or moved. This clearly explains the loss of resolution. During tip reshaping, a short current/voltage spike may have been triggered that induced the change in the sample. This explains why the top part of the image has a different symmetry than the bottom part.

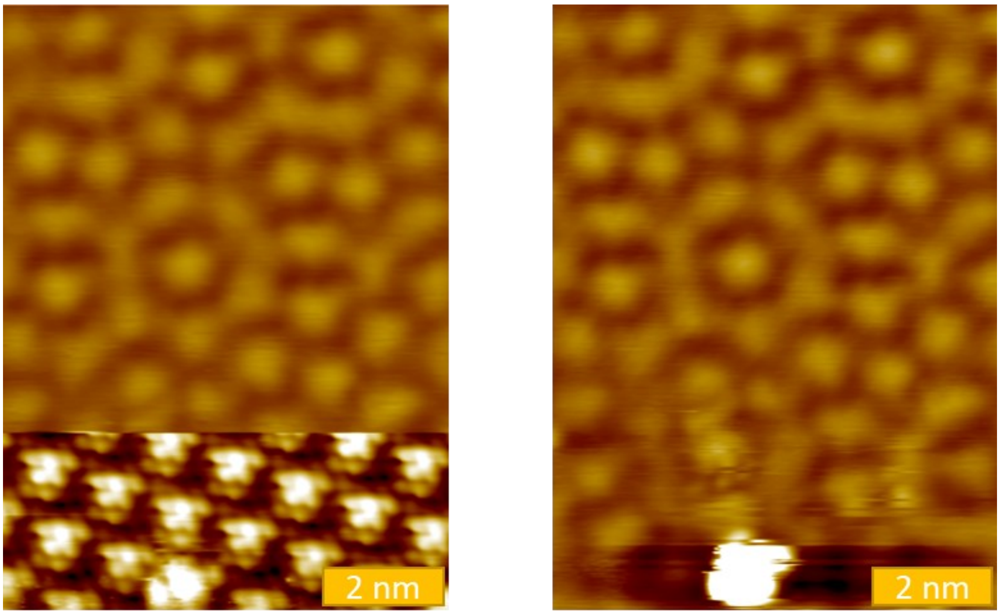

Figure S8. a) STM image of the H state domain (scanning upwards), which abruptly changed to the domain of two interfering CDWs and b) image of the same region taken on the scan down.

A similar process to the one shown in Fig. S8 could have happened also in Fig. S4a. Fig. S4a was scanned on the way up (bottom of the image is taken earlier) and it seems the appearance of the top quarter of the image (where interfering chiralities are present) is a bit different. Since we did not scan this part of the sample before and since the contrast is not very obvious, we cannot claim with confidence when exactly the transition happened, but it is likely that it also happened during the scan.

Influence of the bottom layers on the top layer

To exclude the possibility of interference between electron orders of different chirality in the top two layers, we must look at some examples, where the top and bottom layer CDW are not the same, but we observe no interference effect. The first argument against a multilayer effect is interlayer stacking. It was shown that polarons do not very likely sit directly above polarons in the bottom layer [3,4]. It

was shown previously that in the domain state, the domain walls are shifted, so that the polarons are not aligned between the different domains [5,6]. The polarons from the top and bottom layers would be vertically aligned only if also the domain walls are perfectly aligned, which was shown not to be the case [5]. If – as observed experimentally [4,5] - the domains are shifted with respect to each other, the vertical stacking of polarons varies significantly.

Another argument is that if the interlayer interference was a strong effect, we should see the polarons in the microscope image appearing differently across the image, but they seem to be mostly unperturbed by the layer underneath. In Fig. S8 we present the same argument for the interlayer interference between mirrored superlattices. Here we see that the top layer is partly transformed to the 1H polytype, which has no charge modulation above 75 K (the image was taken at 80 K) and thus appears very flat in STM, and is 'semi-transparent', revealing the charge modulation below. We can clearly see that the superlattice in the layer below has a different direction than in the layer above. Thus, unless there is no domain wall in perfect register with the border between 1H and 1T polytypes (which is very unlikely), there is no interference between the domains of different chirality. Furthermore, if such interlayer interference was strong, it should very likely be observed every time a mirrored domain appears, assuming there is not an identical domain under it, which is again very unlikely.

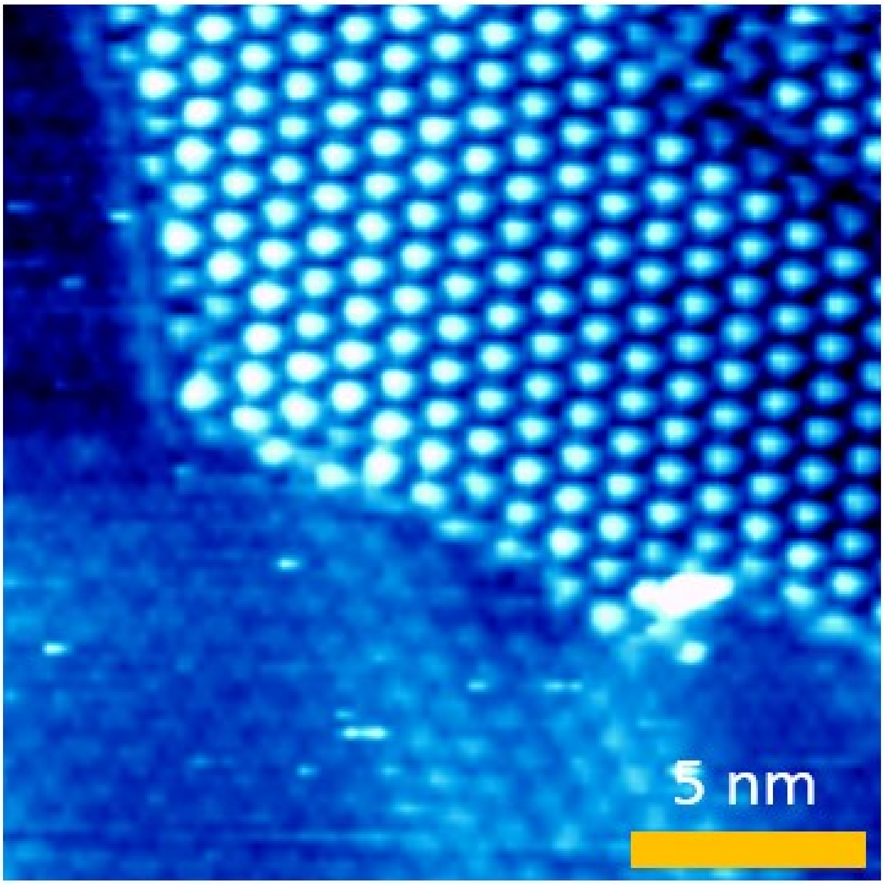

Figure S9. STM image of the H state, where the domains from the bottom layer are also seen. b) partly transformed top layer to the "transparent" 1H polytype. We can see that the CDW modulation in the bottom layer is mirrored with respect to the top layer. In both cases we see no interlayer interference effect.

In order to check for the possibility of similar chirality interference patterns as a result of interaction between the CDW in the top and the second layers of the same 1T polytype, additional STM experiments were performed. During the experiments, the top layer of the material was conditioned in a way to create small (up to 5 nm laterally) single-layer defects (voids in the atomic structure) to be able to directly scan the second layer (Fig. S10a,c). The two CDW lattices (one for L and one for R chirality) were carefully assigned to every domain in the top layer to have the best fit on large-area images. The visible parts of the second layer were fit to the same lattices with no adjustments except for the lateral position. The abovementioned CDW lattices of the second layer were extended towards the expected areas of overlap with the opposite chirality in the top material layer (Fig. S10b,d). The performed analysis has shown, that no chirality interference is observed in the top layer of 1T-TaS2 even when the second layer has an opposite CDW chirality.

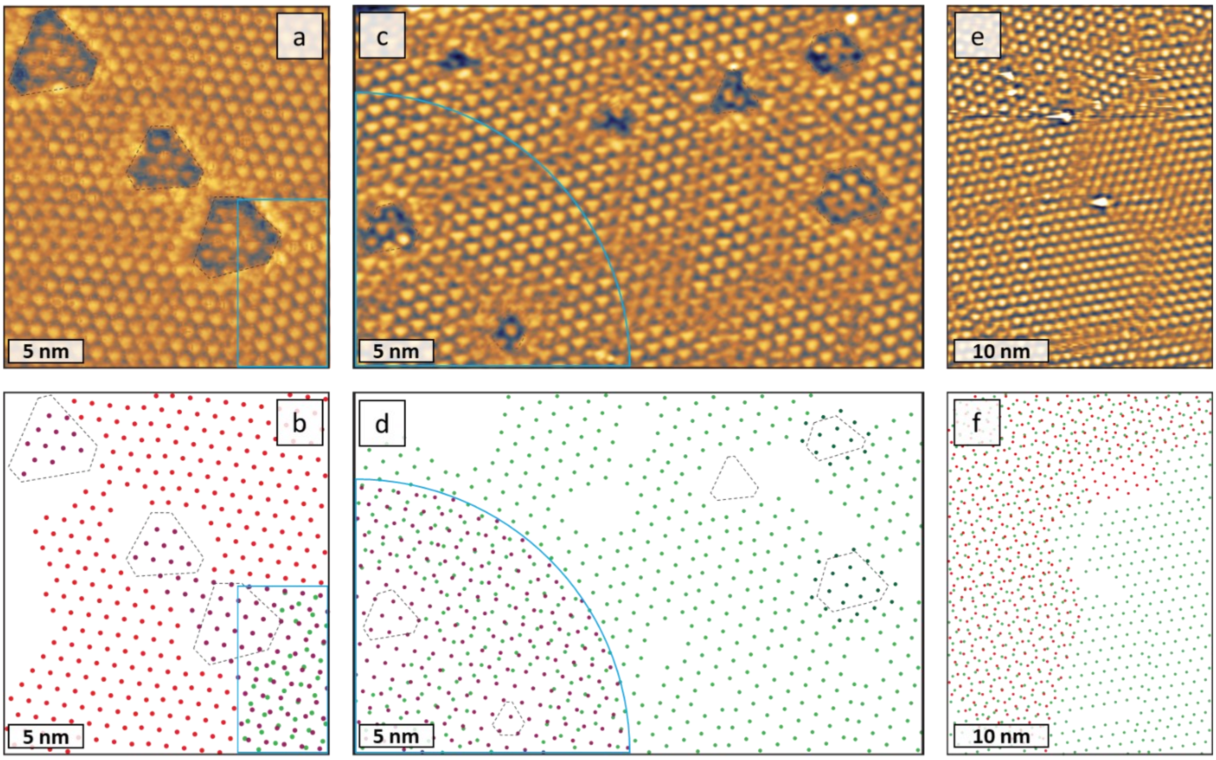

Figure S10. Chiral CDW domains of 1T-TaS_2 observed with STM in the top layer(s). CDW lattices are marked for domains of L (lime) / R (red) chiralities in the top and L (green) / R (violet) chiralities in the second layer. Blue solid lines are marking expected areas of chirality interference (overlap) between layers, gray dashed lines are marking single-layer defects for easy referencing.

a, c) 1T-TaS_2 layers with both (panel a) / single (c) chiralities in the top layer and single (a) / both (c) chiralities in the second layer. No interference pattern is observed.

b, d) CDW lattices fit the experimental data and marking areas of chirality overlap for panels a, c.

e, f) Observed chirality interference pattern and its fit to the CDW domains, partially visible in the top layer.

Interference pattern in 4Hb-TaSe$_2$

We performed measurements also on 4Hb-TaSe$_2$ where 1T and 1H layers are alternating. A transition from an incommensurate to a commensurate $\sqrt{13} \times \sqrt{13}$ charge density wave for 1T layers occurs at around 410 K [7]. This is the same superlattice modulation as in the commensurate state of 1T-TaS$_2$. In 1H layers, the CDW transition to a $\sim 3 \times 3$ CDW appears at around 75 K. The measurements on the 4Hb-TaSe$_2$ were made at 77K, where we expect 1T layers to be in the commensurate CDW phase and 1H layers in the normal phase, just above the phase transition.

Figure S11 a) shows a step between layers with a line scan profile. The top layer is in the 1T phase with a $\sqrt{13} \times \sqrt{13}$ CDW modulation, while the bottom 1H layer has only very weak modulation with a similar periodicity, arising from the 1T layer below the 1H. While scanning the top 1T layer we applied several high-voltage pulses (6V, 5 ms) with the STM tip. We observe an interference pattern 30 nm away from the position where the pulses were applied as shown in Figure S11 b). The interference pattern is stable even after scanning for 4 hours. After moving around different areas, the tip hit an impurity particle on the surface and produced an electrical pulse on the surface. After this, the interference pattern is lost (see Figure S11 c). This is consistent with the observed change in the interference pattern reported in Figure S8 on 1T-TaS$_2$.

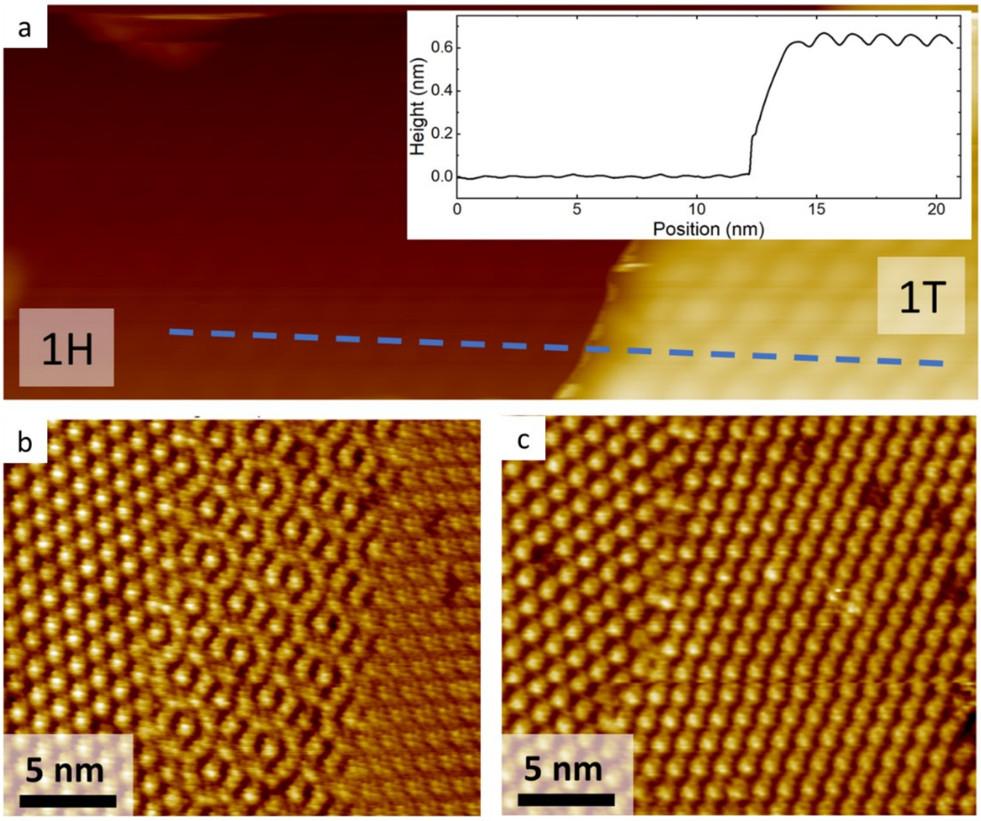

Figure S11. a) Step between layers of the 4Hb-TaSe$_2$: the top layer is 1T with the $\sqrt{13} \times \sqrt{13}$ CDW modulation (right part of the image), while the bottom 1H layer does not exhibit significant modulation (left part of the image). Inset shows a line profile across the step between layers. b) Interference pattern observed on the 1T top layer after several strong electrical pulses from the tip were applied around 30 nm away. c) Interference pattern is lost after scanning over a piece of dirt that produced an unintended electrical pulse. The scanning area of b) and c) are the same.

The structure of domain walls separating domains of different chirality

Between two mirrored domains, the geometry does not allow a simple straight domain wall. In this case, we can nevertheless expect that the domain wall would take the closest approximation to a straight line, in order to minimize its length and thus its energy [8]. In Fig. S12 we show a few conceptual drawings of domain walls, where we attempt to find domain walls with higher symmetry for one or both domains, while keeping the domain wall straight. We can see that in order to keep the boundary of one domain straight, the other domain must asymmetrically adapt to keep the domain wall of a certain average width (Fig. S12a). Such a domain wall is periodic, and the pattern repeats every 13 superlattice sites. Between the two extreme points, where one domain boundary is perfectly straight and the other one adapts and vice versa, we can create an infinite number of different domain walls, which are distorted on both sides. At the midpoint, there is a number of symmetric domain walls, where domains have symmetrically distorted edges. An important thing to notice is the period on which the two domains overlap, which can be described by a linear combination of 13a* and 13b*. Thus, there are lattice positions which correspond to both superlattices and having such polarons in the domain wall may potentially lower the overall energy of the domain wall and further stabilize it. With this in mind, we can try to construct a few such periodic and symmetric domain walls, which are shown in Fig. S12 b-d.

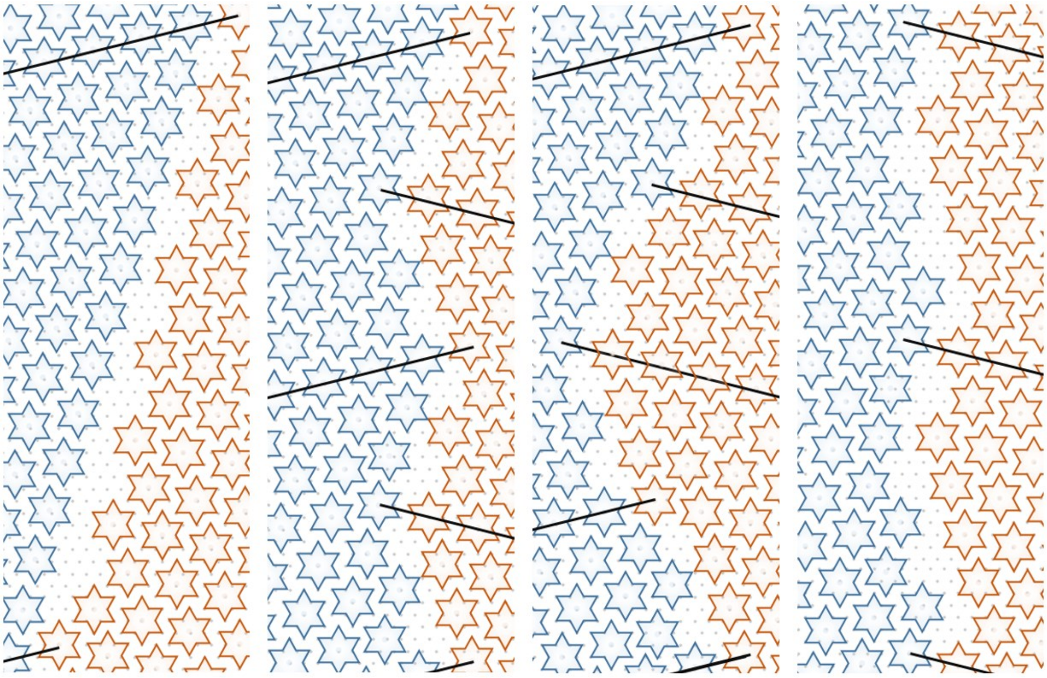

Figure S12. various attempts of creating a domain wall between differently oriented domains, while keeping some form of symmetry. a) One domain has a straight boundary, while the other one completely adapts. b), c) and d) The domain wall is symmetric and aligned with lattice direction. Both domains adapt to keep the symmetry. d) The domain wall is aligned with the lattice direction, but is not symmetric. Black lines are drawn as guidelines to mark where one domain could extend into the other domain.

Appearance of mirror domains for different annealing times.

By changing the simulated annealing time as described in [9] we can influence the probability of the presence of mirror domains. When the annealing time is short, there is a much higher chance to observe both kinds of domains, with an expected average distribution of 50% each. This is dictated by random direction nucleation growth of the domains and simultaneous locking due to the fast annealing times. Additionally, with short annealing times, sometimes even $\sqrt{12} \times \sqrt{12}$ domains are present, which were not previously seen in any experimental case [1]. When annealing the system slowly, we mostly observe only one type of the domains when the low temperature is reached. This implies that the energy of the domain walls between different domains is larger than the energy between the domains of the same type, as they always tend to relax to a group of single-orientation domains when given enough time. This can be confirmed by calculating the final energy of the system in both cases, following the energy calculations as performed in [10]. The source of the difference in energies can be simply pointed out by looking at the figures and consists of two contributions: the domain walls between two domains with the same orientation are in most cases only one polaron spacing thick and perfectly straight, while the domain walls between mirrored domains are thicker and very rugged. In other words, when domains of both orientations are present, the overall coverage of the sample with the $\sqrt{13} \times \sqrt{13}$ superlattice (which is energetically the most favorable for this filling) is smaller, because more of the space is taken by the energetically costly domain walls.

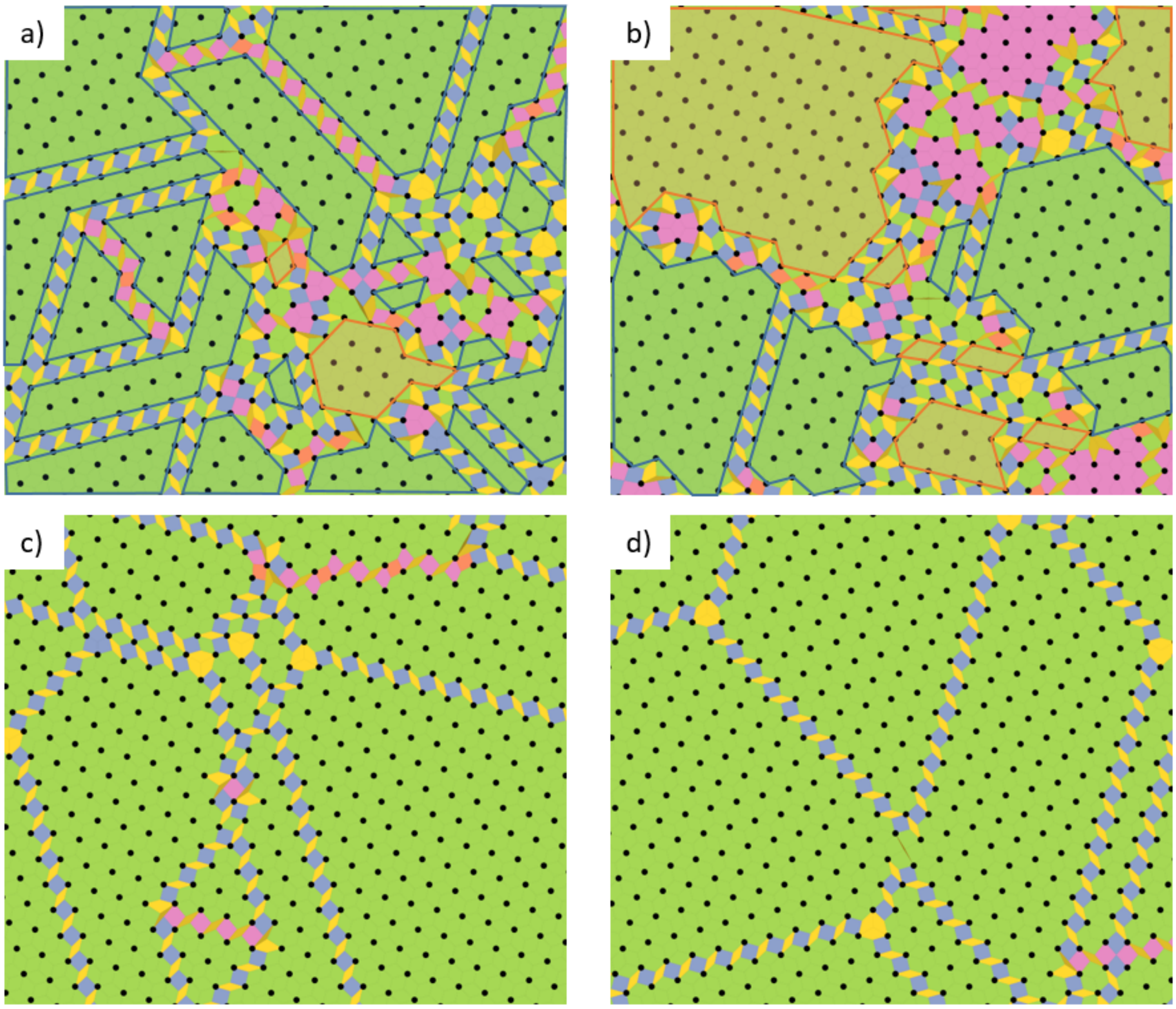

Figure S13. Comparison of different annealing times at f=12.6. a) 90 sweeps, b) 100 sweeps, c) 200 sweeps, d) 300 sweeps. For 100 or less sweeps, we very likely see mirrored domains and sometimes even the √12×√12 domains. When performing longer sweeps, we very rarely observe more than one type of domains. Different domain orientations were marked with blue and orange shapes in a) and b). The √12×√12 domains are tiled with pink. In c) and d) no domains were marked as they are all of the same type.

Direction of the domain walls between the domains with different SL orientations.

Both the experimentally observed and simulated domain walls between mirrored domains are too short to indicate whether they follow a general direction. However, it roughly seems that they tend to align with the atomic lattice direction. To verify this claim and see how longer domain walls would behave, we have employed fixed boundary conditions to the model to simulate two large domains with mirrored superlattice orientations (Fig. S14a). We have used the same model as before, but pinned down the polarons at the two boundaries (highlighted blue, marked with B and C) of the model to form the two distinct chiralities. We put additional unpinned polarons in the empty region between them, and kept the other two boundaries (highlighted yellow, marked with A) of the model periodic. Then, we observed the ordering of the polarons through the same annealing process as originally [10].

As the observed experimental samples are too small to calculate the appropriate filling (number of polarons per number of lattice sites) for this state, we used the filling of the C state (13 sites per polaron) as a basis and simulated the behavior for relatively large deviations (± 0.4) from this filling. The observed filling for the H state is 12.6, which is covered in this range. The filling close around 13 shows only one domain wall and a small deviation of filling does not affect the results qualitatively. However for larger deviations (> 0.1), more domains and domain walls start forming, and a domains form over all of the available area. We stick to the fillings where only one domain wall appears (close to 13) for easier analysis of the domain wall direction. What we can see is that the domain wall in all cases aligns very close to the lattice direction, making the domains almost symmetrical. A potential problem arises from periodic boundary conditions for two of the unpinned borders (top and bottom borders on Fig. S14a), which define the direction of the shortest possible domain wall. In our case, this shortest possible domain wall follows the lattice direction, making it unclear, whether the domain wall tends to align with the lattice direction, or simply looks for the shortest path. In order to clarify that this does not happen due to the periodic boundary conditions, we shifted the boundary in the model (Fig. S14b)). Doing this, the shortest possible domain wall was not anymore along the lattice direction. With the new boundary conditions, the annealing did not result in one simple domain wall, as it did in the case shown in Fig. S14a). In this case we always arrive to a minimum, which is a messy mixture of domains with different orientation and even with some 12×12 domains. The result is the same for a variety of fillings (shown in Fig. S15 and S16). It appears that this kind of the domain wall is more energetically costly when it does not follow the lattice direction and thus tries to relax its energy by breaking into smaller pieces through formation of many smaller domains. We can assume that the same physical laws govern the formation of domain walls between mirrored domains, as they do in formation of the walls between two domains of the same chirality. It is generally accepted that the free energy of the domain walls includes a linear contribution of the length of the domain wall, which forces the domain walls to form straight lines, in order to achieve the lowest energy state [8]. We conclude that the walls between domains with different orientation tend to orient along the direction of the atomic lattice, as this is the way to lower their energy.

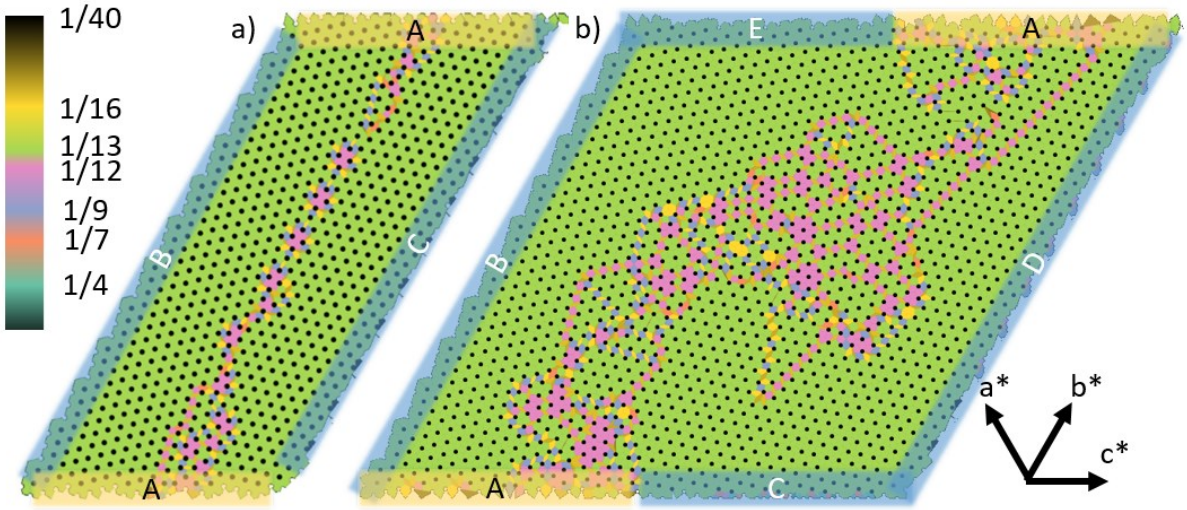

Figure S14. (a) Simulation of the polaron ordering with PBC only in the vertical direction (borders are marked with A), whereas on the left and right borders the polarons were pinned, to fix the two different superlattice orientations (borders B and C). (b) Similar as (a), but with shifted borders to model the domain wall which is not parallel to the lattice direction.

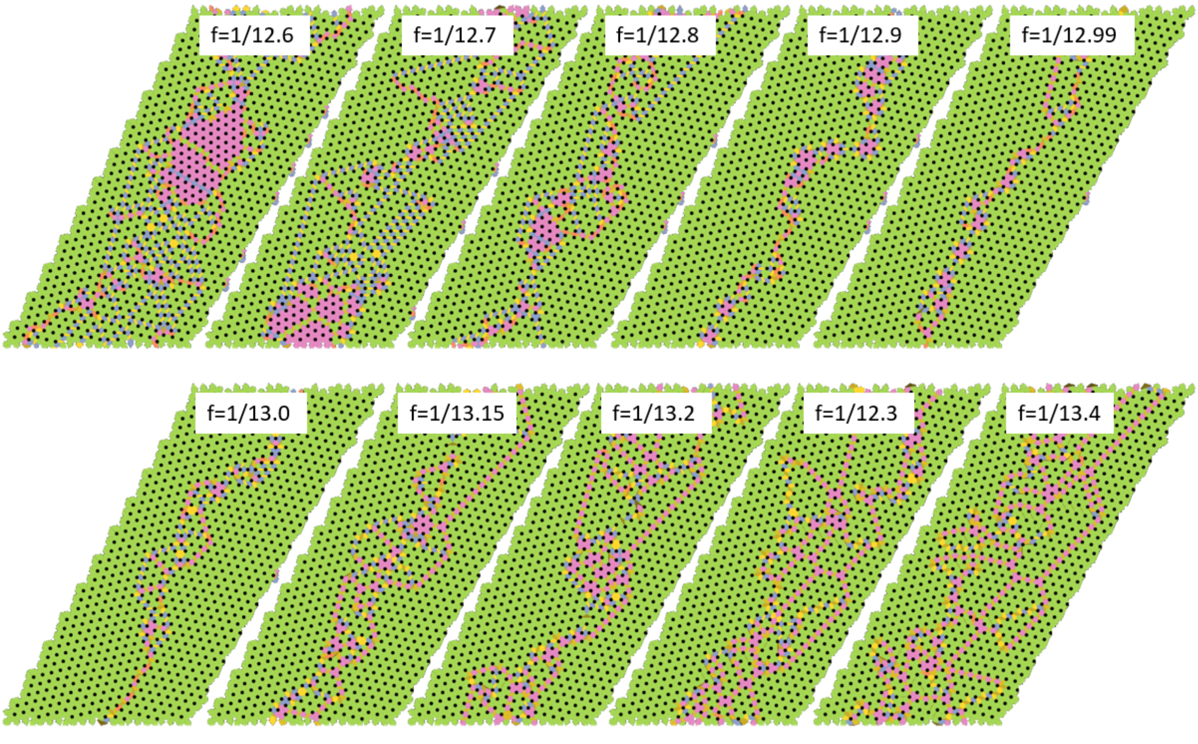

Figure S15. (a) Domain walls between mirrored domains for different fillings. For fillings close to 1/13 the domain wall between the mirrored domains is close to straight and rarely some other domains appear. For larger deviations, the area becomes covered with various domains.

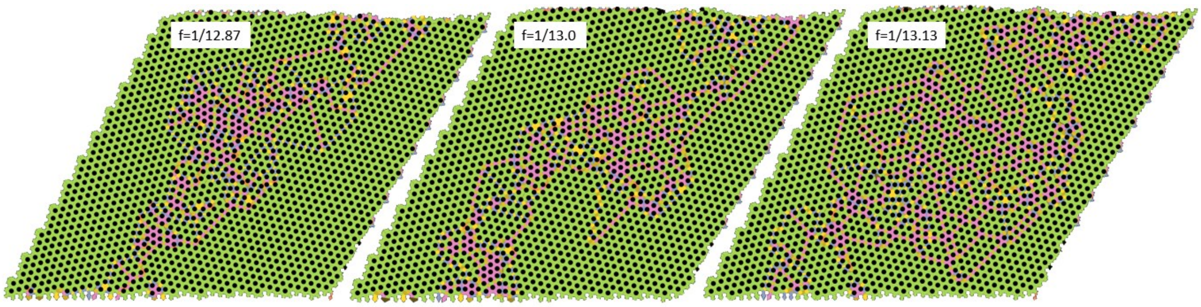

Figure S16. Domain walls between mirrored domains with shifted periodic boundary conditions for different fillings. We observe no conceptual difference of the domain wall formation when changing the filling of the system. We do not observe a single domain wall for any filling.

Modelling of the periodic lattice distortion in the double CDW state

To obtain a qualitative picture of the atomic lattice displacements in the superposition (S) state, we consider a model consisting of atoms that interact via Lennard-Jones potential and are subject to an underlying CDW potential. The periodic lattice distortion of the David star unit cells [11–13] is generally understood as the displacement of the outer twelve atoms towards the thirteenth atom in the center of the star. We assume that the Ta atoms interact via the Lennard-jones potential $V(r) = 4\varepsilon\left[\left(\frac{\sigma}{r}\right)^{12} - \left(\frac{\sigma}{r}\right)^{6}\right]$. Here r denotes the distance between two interacting particles, σ is the distance at which the potential energy is zero and ε is the depth of the potential. The interacting atoms on a 2D surface naturally form the energetically most favorable triangular lattice.

Next, we subject these particles to an external potential in form of the $\sqrt{13} \times \sqrt{13}$ CDW superlattice and perform a Monte-Carlo simulation to observe the formations that the particles will take to minimize the energy. We start with the single-direction CDW. The equation for minimizing the energy of the j-th particle in a left-handed CDW potential can be written as

$$V(r) = 4\sum_{i\neq j}\left[\left(\frac{\sigma}{r_i - r_j}\right)^{12} - \left(\frac{\sigma}{r_i - r_j}\right)^{6}\right] + U\,Re\left(\sum_{d\in\{1,2,3\}} e^{i\overrightarrow{k_d^{+}}\overrightarrow{r_j}}\right),$$

where the first term sums over all of the particles i and calculates the pairwise potential energy between i-th and j-th particle. The second term adds the outside potential energy of the CDW. We show the different potentials in the Fig. S17. Note that in some cases we superimpose also the atomic lattice, for better understanding of the period of the potential.

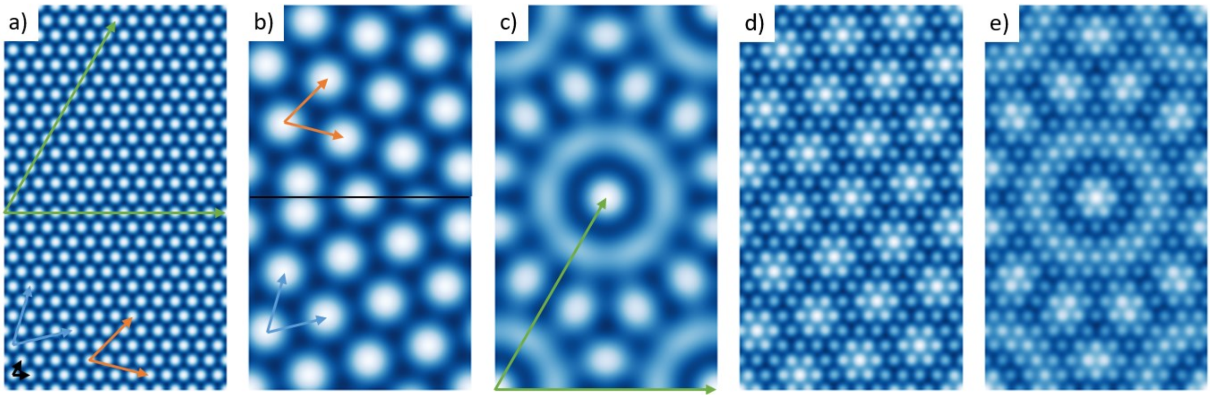

Figure S17. Different shapes of the potential as a sum of cosine terms. All figures are in the same scale. a) The atomic lattice (here the wavevectors k are taken to be the atomic lattice vectors), b) right (top) and left (bottom) handed CDW lattices, c) double CDW lattice (sum of both lattices in b), d) left-handed CDW lattice and atomic lattice (sum of a and b) and e) double CDW with atomic lattice modulation (sum of a, and c). The arrows Show the wavelengths of different waves: Black arrows show the a and b vectors of the atomic unit cell. Blue and orange arrows show the left and right handed CDW vectors and are equal to 3a+b and 4a-b respectively with their 60 degree equivalents. Green arrows show the size of the unit cell in the double CDW, which equals to 13a and 13b. The atomic lattice is in this case assumed perfectly unperturbed and is shown in the figures just as a reference and not used in the further calculations.

In Fig. S18, we show results of the Monte-Carlo simulation for a single CDW potnential with an exaggerated $U = 100$, where the displacements are clearly visible. We can see the formation of a $\sqrt{13} \times \sqrt{13}$ superlattice, where unexpectedly we do not observe the movement of 12 atoms towards the central atom, but only the inner 6 atoms of the expected David-star are displaced, while the outer 6 stay at their initial positions. This makes sense, considering the outer six atoms are sitting on a potential energy saddle, as opposed to the inner six atoms, which move towards the center of the valley in which they are located. Eventually, with an absurdly large U, the outer atoms move as well, but this case is not considered to be physically relevant anymore.

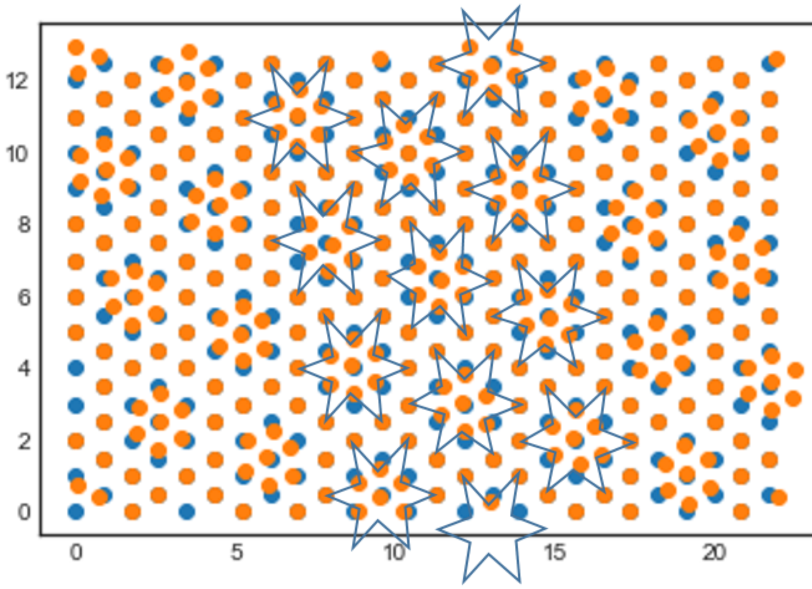

Figure S18. A result of a Monte-Carlo simulation for the particles interacting via Lennard-Jones potential, in an outside potential, which resembles the left handed $\sqrt{13} \times \sqrt{13}$ CDW superlattice (as shown in Fig S14 b). The blue and orange dots represent the equilibrium atomic positions and the displaced positions respectively.

We perform the same simulation using the potential energy of both CDW chiralities (as shown in Fig. S17g). Here we slightly change the equation to add the contribution of both directions of CDW.

$$V(r) = 4\sum_{i\neq j}\left[\left(\frac{\sigma}{r_i - r_j}\right)^{12} - \left(\frac{\sigma}{r_i - r_j}\right)^{6}\right] + U\,Re\left(\frac{1}{2}\sum_{m\in\{+,-\}}\sum_{d\in\{1,2,3\}} e^{i\overrightarrow{k_d^m}\overrightarrow{r_j}}\right),$$

We also normalize the outside potential energy by a factor of ½, as it now consists of two different contributions. The atomic displacements in this case follow a different pattern, which also closely resembles the pattern of the outside potential and is shown in Fig. S19.

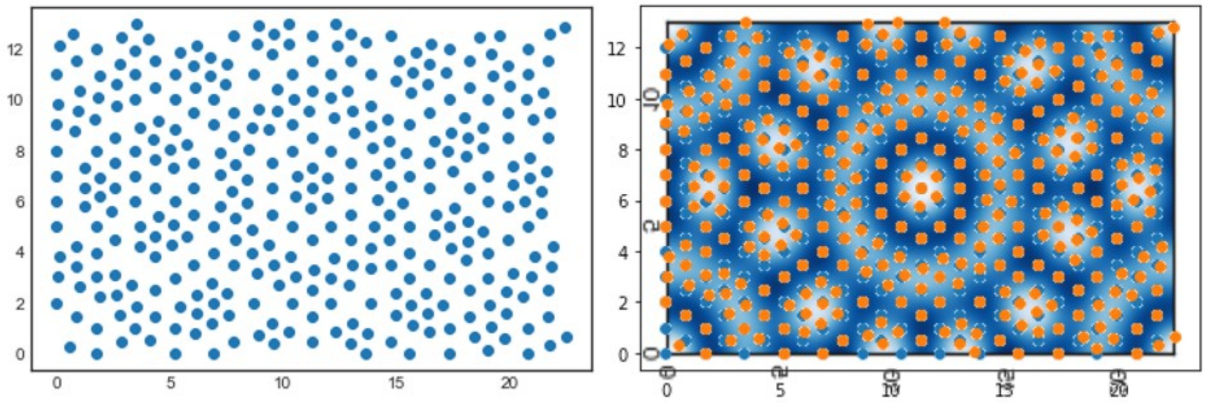

Figure S19. A result of a Monte-Carlo simulation for the particles interacting via Lennard-Jones potential, in an outside potential, which resembles the sum of the left and right handed $\sqrt{13} \times \sqrt{13}$ CDW superlattices. a) the displaced atoms, b) equilibrium and displaced atoms in blue and orange respectively, drawn on top of the double CDW potential energy surface.

To understand the displacements in the interfering CDW better, we compare them to a direct sum of displacements in two separate cases for the L and R CDW direction. For a clearer picture, we write down the atomic displacements in a vector form, assuming perfect atomic displacements (each atom is displaced by exactly the same value in one of the high-symmetry directions – towards the central atom of the star). By comparing the vector displacement with the Monte-Carlo simulation, one can see the best correspondence when we only displace the inner 6 atoms of the star, both for the single direction as well as for the double CDW. The simulated lattice distortions of the double CDW thus simply look like a sum of the displacements in both CDW directions.

We choose three different approaches to the atom displacements within a star: 1) all atoms are displaced equally towards the center of the star (Fig. S20), 2) The outer atoms are displaced twice as

much as the inner atoms (this way the atoms in the star are at equal distances to their neighbors), (Fig. S21) and 3) only the inner 6 atoms in the star are displaced, to get the hexagonal shapes, similar to the ones obtained by the Monte-Carlo simulations (Fig. S22).

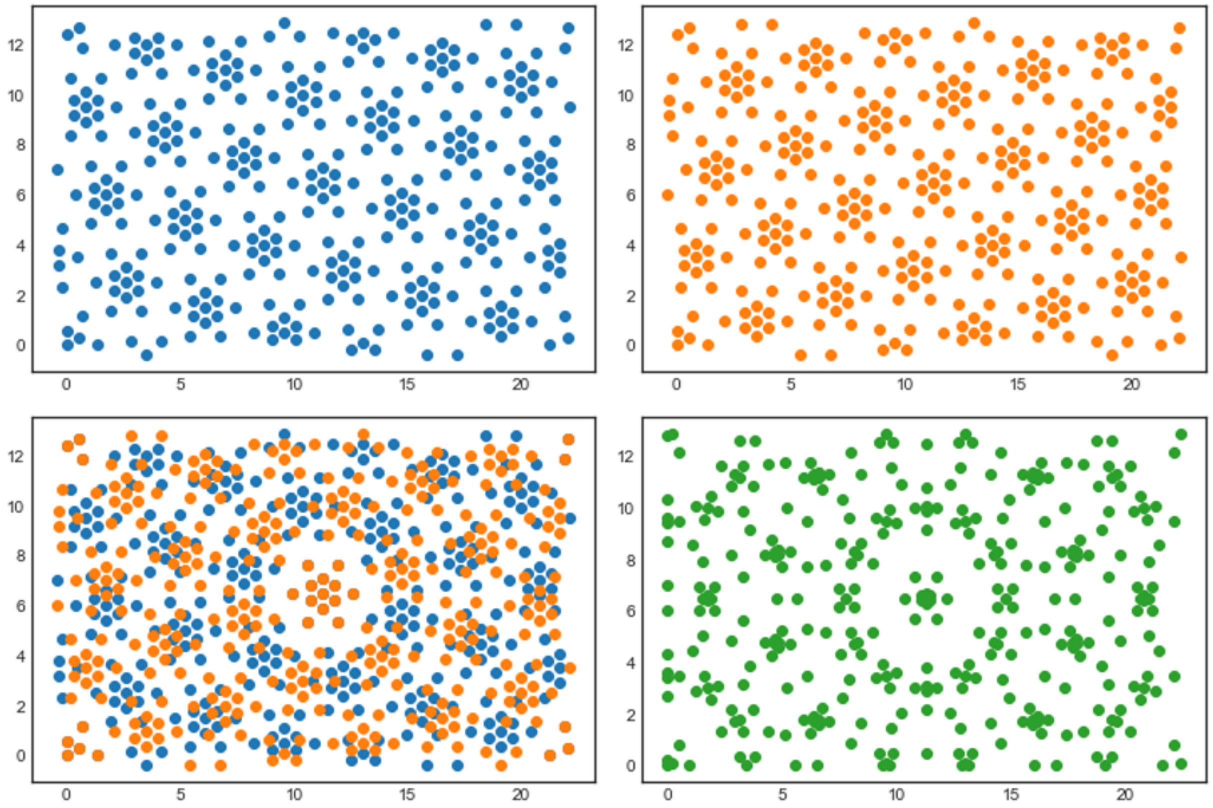

Figure S20. Vector displacements of the atoms, where all 12 atoms are equally displaced towards the center atom, with the exaggerated displacements of 0.4 atomic unit cells. a) left-handed superlattice, b) right-handed superlattice, c) both left and right handed superlattices shown on the same plot and d) a sum of the displacements, calculated for each atom.

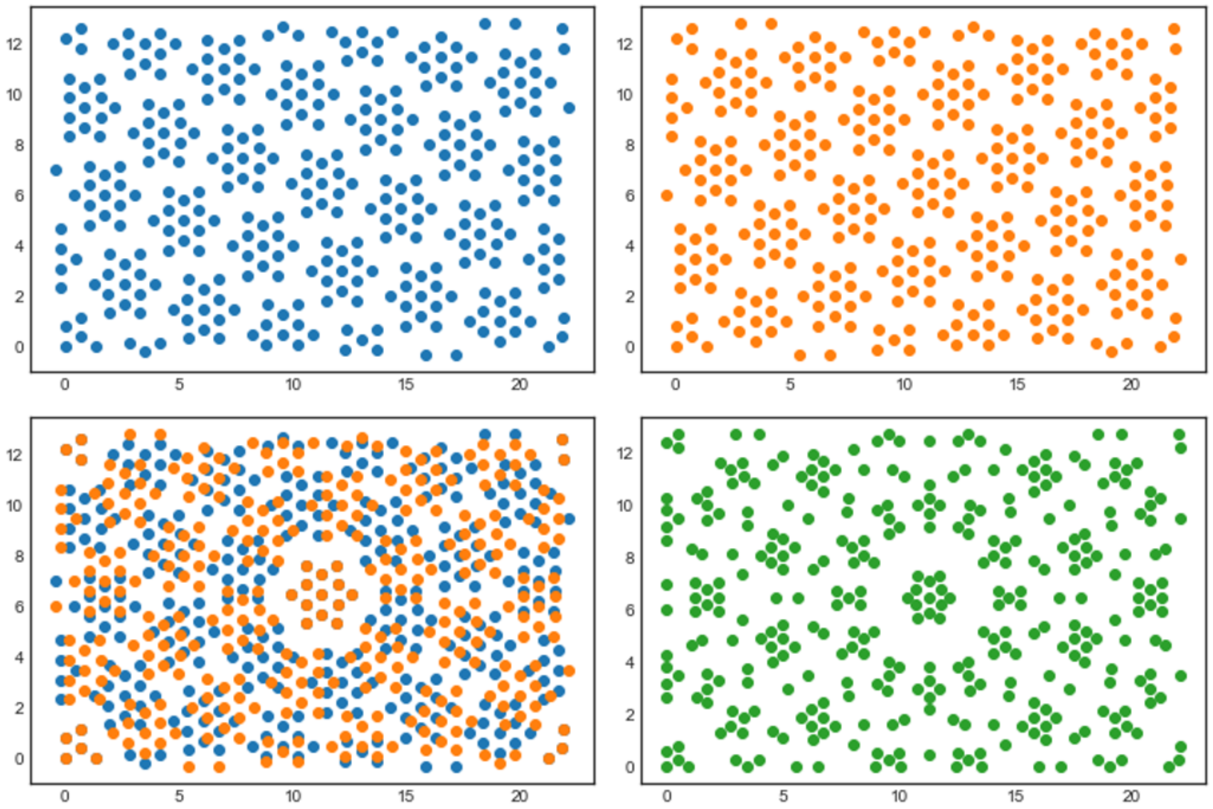

Figure S21. Vector displacements of the atoms, where the outer 6 atoms in the star are displaced twice as much as the inner atoms, for 0.4 and 0.2 atomic unit cells respectively. a) left-handed superlattice, b) right-handed superlattice, c) both left and right handed superlattices shown on the same plot and d) a sum of the displacements, calculated for each atom.

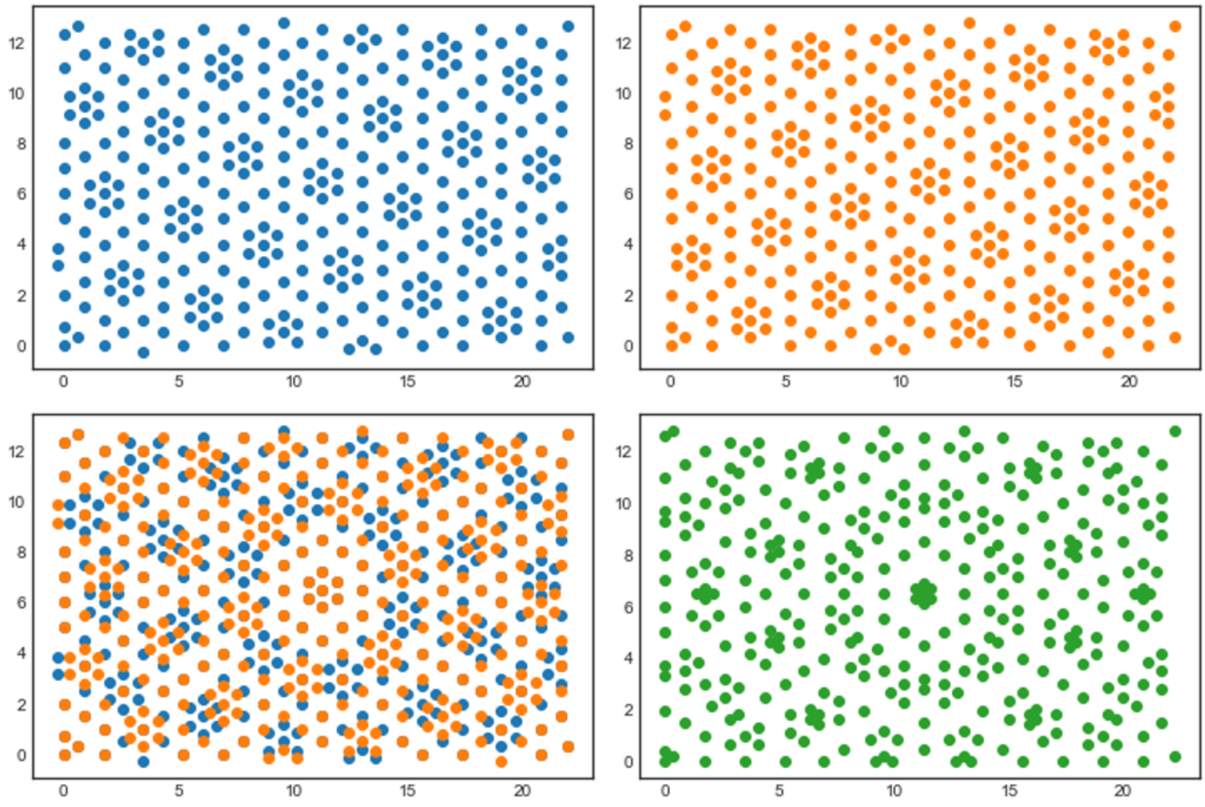

Figure S22. Vector displacements of the atoms, where only the inner 6 atoms of the star are displaced by 0.4 atomic unit cells towards the center atom, but the outer 6 stay unperturbed. a) left-handed superlattice, b) right-handed superlattice, c) both left and right handed superlattices shown on the same plot and d) a sum of the displacements, calculated for each atom.

In Fig. S23 we plot together the Monte-Carlo simulation (blue) and the vector displacement (green). By closely examining the images, we can see that the pattern is indeed very similar.

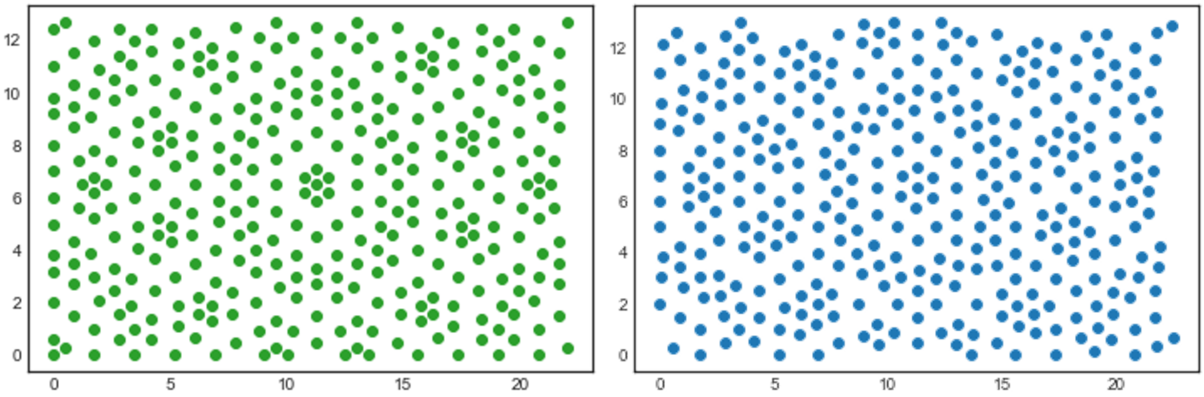

Figure S23. Comparison of the atomic displacements calculated as a vector sum of the two CDWs (the vector displacements are 0.3 atomic units for each CDW direction), shown in green, and the atomic displacements as obtained with the Monte-Carlo simulation, shown in blue.

[1] J. Ravnik, M. Diego, Y. Gerasimenko, Y. Vaskivskyi, I. Vaskivskyi, T. Mertelj, J. Vodeb, and D. Mihailovic, Nature Communications **12**, 1 (2021).

[2] Y. A. Gerasimenko, I. Vaskivskyi, M. Litskevich, J. Ravnik, J. Vodeb, M. Diego, V. Kabanov, and D. Mihailovic, Nature Materials **18**, 1078 (2019).

[3] Q. Stahl, M. Kusch, F. Heinsch, G. Garbarino, N. Kretzschmar, K. Hanff, K. Rossnagel, J. Geck, and T. Ritschel, Nature Communications **11**, 1 (2020).

[4] T. Ritschel, J. Trinckauf, K. Koepernik, B. Büchner, M. v Zimmermann, H. Berger, Y. Joe, P. Abbamonte, and J. Geck, Nature Physics **11**, 328 (2015).

[5] L. Ma, C. Ye, Y. Yu, X. F. Lu, X. Niu, S. Kim, D. Feng, D. Tománek, Y.-W. Son, X. H. Chen, and others, Nature Communications **7**, 10956 (2016).

[6] D. Cho, S. Cheon, K.-S. Kim, S.-H. Lee, Y.-H. Cho, S.-W. Cheong, and H. W. Yeom, Nature Communications **7**, 10453 (2016).

[7] F. Di Salvo, D. Moncton, J. Wilson, and S. Mahajan, Physical Review B **14**, 1543 (1976).

[8] J. Villain, Surface Science **97**, 219 (1980).

[9] J. Vodeb, V. V. Kabanov, Y. A. Gerasimenko, I. Vaskivskyi, J. Ravnik, and D. Mihailovic, Journal of Superconductivity and Novel Magnetism **32**, 3057 (2019).

[10] J. Vodeb, V. V. Kabanov, Y. A. Gerasimenko, J. Ravnik, M. A. van Midden, E. Zupanic, P. Sutar, D. Mihailovic, and others, New Journal of Physics **21**, 083001 (2019).

[11] R. Brouwer and F. Jellinek, Physica B+ C **99**, 51 (1980).

[12] C. Sohrt, A. Stange, M. Bauer, and K. Rossnagel, Faraday Discussions **171**, 243 (2014).

[13] L. Stojchevska, I. Vaskivskyi, T. Mertelj, P. Kusar, D. Svetin, S. Brazovskii, and D. Mihailovic, Science **344**, 177 (2014).
